# The evolutionary modifications of a GoLoco motif in the AGS protein facilitate micromere formation in the sea urchin embryo

**DOI:** 10.1101/2024.06.30.601440

**Published:** 2024-07-02

**Authors:** Natsuko Emura, Florence D.M. Wavreil, Annaliese Fries, Mamiko Yajima

**Affiliations:** MCB Department, Brown University, 185 Meeting Street, BOX-GL277, Providence, RI 02912, USA

**Keywords:** AGS, micromere formation, organizer function, embryonic development, sea urchin

## Abstract

The evolutionary introduction of asymmetric cell division (ACD) into the developmental program facilitates the formation of a new cell type, contributing to developmental diversity and, eventually, to species diversification. The micromere of the sea urchin embryo may serve as one of those examples: An ACD at the 16-cell stage forms micromeres unique to echinoids among echinoderms. We previously reported that a polarity factor, Activator of G-protein Signaling (AGS), plays a crucial role in micromere formation. However, AGS and its associated ACD factors are present in all echinoderms and across most metazoans, leaving a question of what evolutionary modification of AGS protein or its surrounding molecular environment contributed to the evolutionary acquisition of micromeres only in echinoids. In this study, we learned that the GoLoco motifs at the AGS C-terminus play critical roles in regulating micromere formation in sea urchin embryos. Further, other echinoderms’ AGS or chimeric AGS that contain the C-terminus of AGS orthologs from various organisms showed varied localization and function in micromere formation. In contrast, the sea star or the pencil urchin orthologs of other ACD factors were consistently localized at the vegetal cortex in the sea urchin embryo, suggesting that AGS may be a unique variable factor that facilitates ACD diversity among echinoderms. Consistently, sea urchin AGS appears to facilitate micromere-like cell formation and accelerate the enrichment timing of the germline factor Vasa during early embryogenesis of the pencil urchin, an ancestral type of sea urchin. Based on these observations, we propose that the molecular evolution of a single polarity factor facilitates ACD diversity while preserving the core ACD machinery among echinoderms and beyond during evolution.

## Introduction

Asymmetric cell division (ACD) is a developmental process that facilitates cell fate diversification by distributing fate determinants differently between daughter cells. It is an essential process for multicellular organisms since it creates distinct cell types, leading to different tissues in an organism. For example, in *Drosophila,* embryonic neuroblasts divide asymmetrically to produce apical self-renewing neuroblasts and basal ganglion mother cells (Bate, 1978; Doe, 2008; Doe et al., 1988; Hartenstein & Campos-Ortega, 1984). In *C. elegans,* the zygote divides asymmetrically to form a large anterior and a small posterior blastomere with distinct cell fates (Schnabel et al., 1996; Sulston et al., 1983; Watts et al., 1996). In mammals, neuroepithelial cells undergo ACD to produce apical self-renewing stem cells as well as basal neural progenitor cells (Chenn & McConnell, 1995; Haydar et al., 2003; Konno et al., 2008; Noctor et al., 2004). A set of polarity factors conserved across phyla regulates these highly organized ACD processes. However, the timing and location of such controlled ACD often occur randomly, even within the same phylum, providing uniqueness to the developmental program of each species. Therefore, we hypothesize that drastic changes in the ACD machinery are unnecessary. Instead, a slight modification in the ACD machinery may drive the formation of a new cell type and the change in the developmental program, contributing to species diversification in the process of evolution.

**Figure 1.**
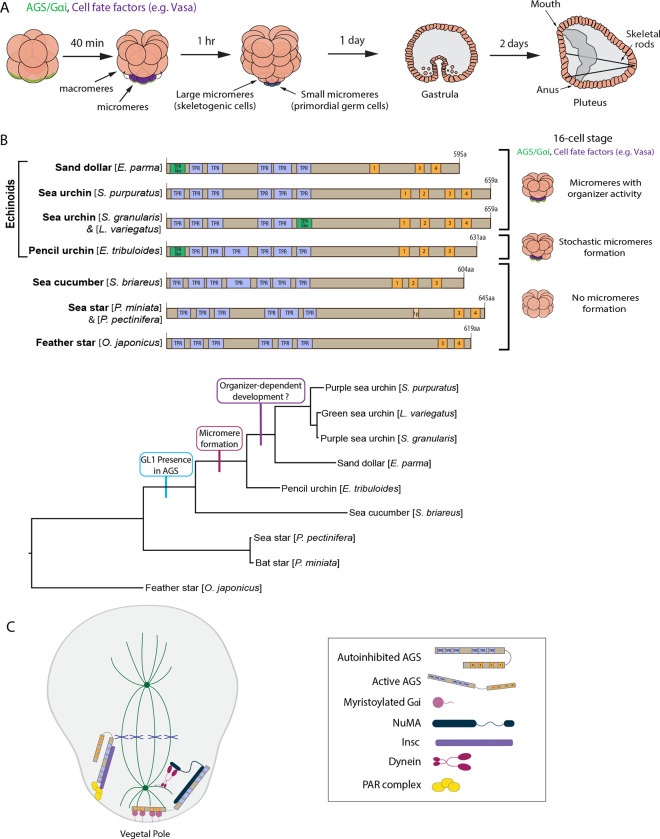
The evolutionary modification of the SpAGS protein corresponds to the introduction of micromeres and inductive signaling during echinoid diversification. **A,** Schema depicting sea urchin embryonic development from 8-cell stage to pluteus. Green represents the colocalization of AGS and Gαi at the vegetal cortex, and purple represents the early segregation of fate factors such as Vasa. **B,** Comparative diagrams of predicted motifs of each echinoderm AGS protein, based on NCBI blast search results for AGS sequences. Conserved TPR motifs are indicated in blue, and GL motifs in orange. Green shows TPR-like motifs, which contain several amino acid changes. Lighter colors represent partial GL motifs. See Fig. S2 for each echinoderm AGS sequence. The tree depicts SpAGS evolution among echinoderms with the introduction of the GL1 motif and micromeres. **C,** Working model of AGS mechanism in ACD based on fly and mammalian models.

In this study, we use echinoderm embryos as a model system to test this hypothesis. Echinoderms are basal deuterostomes and include sea urchins, sea stars, and sea cucumbers, among others. In the well-studied echinoderm models, the sea urchin and sea star embryos, the first ACD or symmetry break occurs at the 8-cell stage, where a horizontal cell division separates animal and vegetal blastomeres that contribute to ectoderm and endomesoderm lineages, respectively (Fig. 1A). However, in the next cell cycle at the 16-cell stage, the sea urchin embryo undergoes an apparent unequal cell division, producing four micromeres at the vegetal pole. In contrast, the sea star embryo undergoes seemingly an equal cell division (Fig. 1B).

The micromere formation in the sea urchin embryo is a highly controlled ACD event since this cell lineage undergoes autonomous cell specification and functions as organizers as soon as it is formed at the 16-cell stage (Horstadius, 1928; Ransick & Davidson, 1993). For example, micromeres autonomously divide asymmetrically again to produce large and small micromeres that are committed to two specific lineages of skeletogenic cells and the germline, respectively, at the 32-cell stage (Okazaki, 1975; Yajima & Wessel, 2011). This early segregation of the germline is unique to sea urchins among echinoderms (Juliano et al., 2009; Fresques et al., 2016). Further, micromeres induce endomesoderm specification (e.g., gastrulation) even when they are placed in the ectopic region of the embryo, such as the animal cap, suggesting they function as a major signaling center in this embryo (Horstadius, 1928; Ransick & Davidson, 1993). The removal of sea urchin micromeres results in compromised or delayed endomesoderm development and compensatory upregulation of a germline factor, Vasa, to presumably start over the developmental program (Ransick & Davidson, 1993; Voronina et al., 2008).

In contrast, other echinoderms undergo minor unequal cell divisions during early embryogenesis, yet they may not be linked to specific cell fate or function. For example, in sea star embryos, the removal of smaller cells does not impact embryonic patterning, and unequal cell divisions appear to be not necessarily linked to specific cell fate regulation or function (Barone et al., 2022). Similarly, even in sea urchin embryos, the non-micromere blastomeres at the 16-cell stage can change the cell fate in response to external cues, including the signaling from micromeres. Recent studies using single-cell RNA-seq analysis further support these observations by demonstrating the earlier molecular segregation of the micromere lineage, while other cell lineages appear to undergo more regulative development (Foster et al., 2019; Massri et al., 2021).

Fossil records and phylogenetic tree analysis suggest that sea urchins diverged relatively later from the common ancestor of echinoderms (Bottjer et al., 2006; Wada and Sato et al., 1994). Since micromeres are unique to echinoids (sea urchins, sand dollars, pencil urchins), they are considered to have emerged later during sea urchin diversification, which has dramatically changed the developmental style in the sea urchin embryo (Emura and Yajima, 2022). To understand how this unique lineage has emerged during evolution, we previously identified the Activator of G-protein signaling (AGS) (Pins in *Drosophila*; LGN in mammals) as a major regulator of micromere formation (Poon et al., 2019). AGS is a polarity factor and plays a role in the ACD of many organisms (reviewed by di Pietro et al., 2016; Kotak, 2019; Rose & Gonczy, 2014; Siller & Doe, 2009; Wavreil & Yajima, 2020; Yu et al., 2006). In the sea urchin (*S. purpuratus;* Sp), SpAGS localizes to the vegetal cortex before and during micromere formation, and its knockdown inhibits micromere formation (Poon et al., 2019). On the other hand, in the sea star (*P. miniata;* Pm), PmAGS shows no cortical localization nor any significant role in ACD during early embryogenesis. Furthermore, the pencil urchin (*E. tribuloides;* Et) is an ancestral type of the sea urchin and diverged around 252 million years ago, which is located between the sea star and the sea urchin. The Et embryo exhibits an intermediate developmental program of the sea urchins and sea stars. It stochastically forms zero to four micromere-like cells (Fig. 1B). In these embryos, EtAGS localizes to the vegetal cortex only when the embryos form micromere-like cells (Poon et al., 2019), suggesting a close correlation between cortical AGS localization and micromere-like cell formation.

Furthermore, the introduction of sea urchin AGS into sea star embryos induces random unequal cell divisions by recruiting the spindle poles to the cortex (Poon et al., 2019), suggesting that SpAGS facilitates unequal cell divisions even in other echinoderm species. Phylogenetic analysis of AGS orthologs across taxa suggests that AGS orthologs increased the functional motif numbers over the course of evolution, likely allowing additional molecular interactions and mechanisms to modulate ACD in a more nuanced manner in higher-order organisms (Wavreil and Yajima, 2020). Supporting this hypothesis, indeed, prior studies suggest that the higher-order mouse AGS ortholog (LGN) can substitute for its fly ortholog (Pins) in *Drosophila* cells, while the basal-order fly Pins cannot substitute its chick ortholog function in chick , the higher-order organism (Yu et al., 2003; Saadaouri et al., 2017). These observations led us to hypothesize that the molecular evolution of AGS orthologs drives ACD diversity across taxa, contributing to the developmental diversity within each phylum. In this study, through a series of molecular dissection experiments, we demonstrate that the AGS C-terminus is a variable region and creates its functional diversity in ACD control, facilitating the developmental variations among echinoderms. This study provides insight into how the molecular evolution of a single polarity contributes to developmental diversity within each phylum.

## Results

### The N-terminal TPR domain is vital for restricting SpAGS localization and function at the vegetal cortex.

AGS consists of two functional domains: the N-terminus contains tetratricopeptide repeats (TPR), and the C-terminus contains G-protein regulatory motifs (GoLoco, GL) (Bernard et al., 2001). AGS switches between a closed and open structure based on the intramolecular interaction between the TPR and GL motifs (Du & Macara, 2004; Johnston et al., 2009; Nipper et al., 2007; Pan et al., 2013). The TPR motifs regulate protein-protein interaction with various partners such as Inscuteable (Insc) for its proper cortical localization or Nuclear Mitotic Apparatus (NuMA) for its microtubule-pulling force generation. In contrast, the GL motifs interact with the heterotrimeric G-protein subunit Gαi for its anchoring to the cortex (Bowman et al., 2006; Culurgioni et al., 2011; Culurgioni et al., 2018; Du & Macara, 2004; Parmentier et al., 2000; Wang et al., 2011; Yu et al., 2000). Studies investigating AGS mechanisms in fly and mammals reveal that Pins/LGN (AGS orthologs) generally remain in the autoinhibited form in the cell (Du & Macara, 2004; Johnston et al., 2009; Nipper et al., 2007) (Fig. 1C). At the time of ACD, Insc recruits Pins/LGN to the cortex through Gαi. This Gαi-binding releases Pins/LGN from its autoinhibition and allows it to interact with NuMA, which recruits the motor protein dynein to generate pulling forces on the microtubules and facilitate ACD (Bowman et al., 2006; Culurgioni et al., 2011; Izumi et al., 2006; Parmentier et al., 2000; Schaefer et al., 2001; Siller et al., 2006; Williams et al., 2014; Yu et al., 2000; Yuzawa et al., 2011; Zhu et al., 2011a).

**Figure 2.**
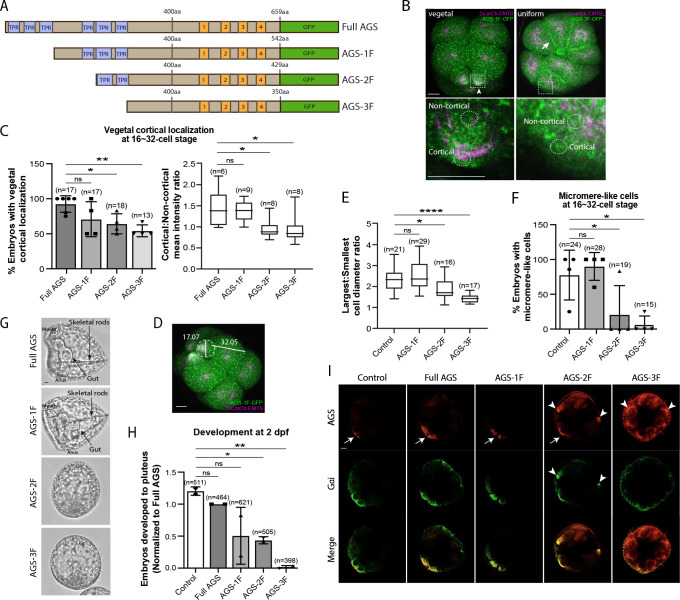
The N-terminal TPR domain restricts SpAGS localization and function at the vegetal cortex. **A**, Design of SpAGS-GFP N-terminal deletion constructs used in this study. TPR motifs are marked in blue, and GL motifs are in orange. **B**, Representative 2D-projection images of the embryo injected with AGS-3F and 2x-mCherry-EMTB, exhibiting vegetal (right panel, arrowhead) and uniform (left panel, arrow) cortical localization. The magnified images below each panel demonstrate how to measure the cortical and non-cortical mean intensities using *ImageJ*, as depicted in the corresponding graph (C). Embryos were injected with 0.15–0.3μg/μl stock of SpAGS-GFP mRNA and 0.5μg/μl stock of 2x-mCherry-EMTB mRNA. Z-stack images were taken at 1μm intervals to cover a layer of the embryo. **C,** % of the embryos with vegetal cortical localization of SpAGS (left) and the ratio of the cortical-to-non-cortical mean intensity (right) at 16~32-cell embryos. Statistical analysis was performed against Full AGS by One-Way ANOVA. **D,** Representative 2D-projection confocal image of a 16-cell stage embryo injected with AGS-1F. The largest cell (macromere) and the smallest cell (micromere) diameters were measured using *ImageJ*. Z-stack images were taken at 1μm intervals to cover a layer of the embryo. **E**, The diameter ratio of the smallest cell (micromere-like cell) over the largest cell (macromere-like cell) was quantified for the embryos injected with the SpAGS mutants or EMTB-only (control). **F**, % of the embryos forming micromere-like cells was scored for each SpAGS mutant and EMTB-only (control). “Micromere formation” is defined as the formation of a group of four cells that are smaller in size and made through a vertical cell division at the vegetal pole at the 16-cell stage. Since none of the AGS-3F-injected embryos formed normal micromeres, “micromere-like cells” were counted based on their vertical cell division, not relative to their size. Statistical analysis was performed against Control by One-Way ANOVA. **G-H** Brightfield images show the representative phenotypes scored in the corresponding graph (H) at 2 dpf. We categorized embryos into three groups, namely, “full development,” with embryos reaching the pluteus stage with complete gut formation and skeleton; “delayed development,” with some gastrulation but no proper skeleton; and “failed gastrulation.” As many of the abnormal-looking embryos fell into the median of the latter two categories, we scored only the embryos reaching full development in the graph. Control represents embryos injected with dye only. Statistical analysis was performed against Control by One-Way ANOVA. **I,** Single Z-slice confocal imaging was used to focus on the vegetal cortex. Embryos were stained with AGS (orange) and Gɑi (green) antibodies. White arrows and arrowheads indicate the signals at the vegetal cortex and ectopic cortical signals, respectively. Images represent over 80% of the embryos observed (n=30 or larger) per group. n indicates the total number of embryos scored. * represents p-value < 0.05, ** p-value < 0.01, and **** p-value <0.0001. Each experiment was performed at least three independent times. Error bars represent standard error. Scale bars=10μm.

To test whether sea urchin (*S. purpuratus*; Sp) AGS functions in ACD similarly to its orthologs, we first investigated the role of its N-terminus by constructing a series of GFP-tagged deletion mutants (Fig. 2A; Fig. S1). AGS-1F is missing the first three TPR motifs, AGS-2F the first four, and AGS-3F the entire TPR domain of SpAGS Open Reading Frame (ORF). The mRNA for these deletion constructs was co-injected with 2x-mCherry-EMTB, a microtubule marker, to visualize the cell cycle phase, spindle location, and orientation. We counted the number of embryos with vegetal cortical localization and conducted a quantitative analysis by measuring the ratio of cortical and non-cortical signal intensity at the 16~32-cell stage (Fig. 2B–C). Embryos injected with full-length SpAGS (Full AGS) or AGS-1F exhibited vegetal cortex-specific localization. In contrast, AGS-2F and AGS-3F showed uniform cortical localization (Fig. 2B–C). These results suggest that TPR4–6 is necessary for restricting AGS to the vegetal cortex, whereas TPR1–3 appears to play a less critical role in controlling AGS localization.

In the control and the AGS-1F groups, micromeres were approximately half the size of the macromeres. In contrast, they were three-quarters the size in the AGS-2F group and almost the same size in the AGS-3F group (Fig. 2D–E), resulting in failed micromere formation even in the presence of the endogenous SpAGS (Fig. 2F). In these embryos, we also scored embryonic development at two days post fertilization (2 dpf) when gastrulation occurs. The AGS-1F mutant mostly showed normal development with extended skeletal rods, whereas AGS-2F and AGS-3F dramatically compromised development with incomplete skeleton extension or gut formation (Fig. 2G–H). Since these N-terminal deletions appear to cause a dominant negative phenotype, we did not knock down endogenous SpAGS in these experiments.

These results suggest that the N-terminal TPR domain is necessary to restrict SpAGS localization at the vegetal cortex. The TPR deletion disables AGS mutants from maintaining the autoinhibited form. It may thus induce their binding to Gαi at every cortex and compete out the endogenous SpAGS at the vegetal cortex. Notably, Gαi localization was also recruited to the exact ectopic location as AGS-2F and −3F mutants (Fig. 2I), suggesting that the SpAGS C-terminus is sufficient to control the Gαi localization at the vegetal cortex. Protein sequences of AGS orthologs across echinoderms are almost identical in their N-termini, suggesting that the AGS N-terminus serves as a core functional domain (Fig. S2). In contrast, the AGS C-terminus appears highly variable across echinoderms.

### The C-terminal GL1 motif is essential for SpAGS localization and function in ACD

**Figure 3.**
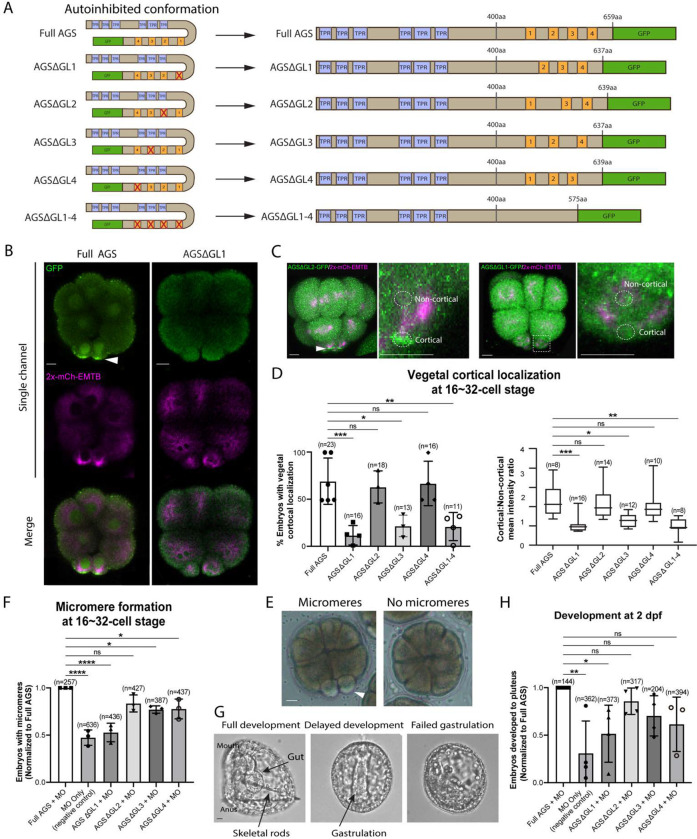
GL1 is essential for vegetal cortical recruitment of SpAGS at the 8~16-cell stage of the sea urchin embryo. **A**, Design of SpAGS-GFP C-terminal deletion mRNAs tested in this study. TPR motifs are marked in blue, and GL motifs are in orange. See Fig. S2 for protein sequence. **B**¸ Single Z-slice confocal imaging was used to focus on the vegetal cortex. Representative embryos injected with SpAGS-GFP or SpAGSΔGL1-GFP are shown. Embryos were injected with 0.3μg/μl stock of SpAGS-mutant-GFP mRNA (green) and 0.5μg/μl stock of 2x-mCherry-EMTB mRNA (magenta). The white arrowhead indicates vegetal cortical localization of AGS-GFP. **C-D**, Representative 2D-projection images of the embryo injected with SpAGS-GFP mRNAs and 2x-mCherry-EMTB, exhibiting vegetal cortical (left panel, AGSΔGL2, arrowhead) and uniform cytoplasmic (right panel, AGSΔGL1) localization. The magnified images right each panel demonstrate how to measure the cortical and non-cortical mean intensities using *ImageJ*, as depicted in the corresponding graph (D). Z-stack images were taken at 1μm intervals to cover a layer of the embryo. % of the embryos that had the GFP signal at the vegetal cortex (left) and the ratio of the cortical-to-non-cortical mean intensity (right) during the 16~32-cell stage were scored in the graphs. Statistical analysis was performed against Full AGS by One-Way ANOVA. **E-F,** Brightfield images show the representative phenotypes scored in the corresponding graph (F) at the 16-cell stage. White arrowhead shows micromeres. Embryos were injected with 0.15μg/μl stock of SpAGS-GFP mRNAs and 0.75mM SpAGS MO. The number of embryos forming micromeres was scored and normalized to that of Full AGS in the graph. Statistical analysis was performed by One-Way ANOVA. **G-H,** Brightfield images show the representative phenotypes scored in the corresponding graph (H) at 2 dpf. Embryos were injected with 0.15μg/μl stock of SpAGS-GFP mRNAs and 0.75mM SpAGS MO. The number of embryos developing to the pluteus stage was scored and normalized to that of Full AGS in the graph. Statistical analysis was performed by One-Way ANOVA. n indicates the total number of embryos scored. * represents p-value < 0.05, ** p-value < 0.01, *** p-value <0.001, and **** p-value <0.0001. Each experiment was performed at least three independent times. Error bars represent standard error. Scale bars=10μm.

To test whether a variable AGS C-terminus creates functional diversity in ACD, we made a series of GFP-tagged C-terminus deletion SpAGS mutants that are missing different GL motifs (Fig. 3A). SpAGS mutants missing GL1 (ΔGL1), GL3 (ΔGL3) or all GL motifs (ΔGL1–4) failed to localize at the vegetal cortex compared to the Full AGS control (Fig. 3B–D), suggesting that GL1 and GL3 are essential for cortical localization of AGS. Next, we knocked down endogenous AGS by morpholino antisense oligonucleotides (MO), which was previously validated for the specificity (Poon et al., 2019). We tested whether these deletion mutants could rescue micromere formation (Fig. 3E). The GL1 deletion significantly reduced micromere formation. In contrast, the GL2, GL3, or GL4 deletion showed no or little significant difference in micromere formation compared to the Full AGS control group (Fig. 3F). Consequently, the GL1 deletion showed significant disruption in embryonic development at 2 dpf, likely due to a lack of micromeres’ inductive signaling at the 16-cell stage (Fig. 3G–H).

These results suggest GL1 is critical for both AGS localization and function at the vegetal cortex for micromere formation. GL3 and GL4 are important for intramolecular binding to the TPR domain in other organisms, which may impact the proper open-close control of AGS protein (Du & Macara, 2004; Johnston et al., 2009; Nipper et al., 2007; Pan et al., 2013).

### The position of GL1 is important for SpAGS function in ACD

**Figure 4.**
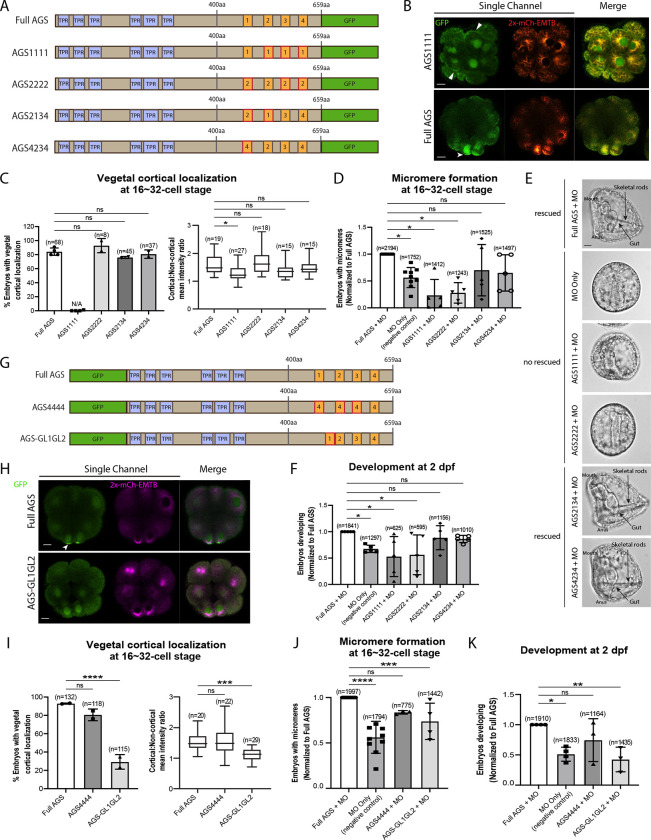
The position of GL1 and the sequences of GL3 and GL4 are important for SpAGS localization and function. **A**, Design of SpAGS-GFP C-terminal mutant constructs tested in this study. TPR motifs are marked in blue, and GL motifs are in orange. Red boxes show interchanged GL motifs. **B**¸ Single Z-slice confocal images of sea urchin embryos at the 8~16-cell stage showing localization of SpAGS1111-GFP mutant. Embryos were injected with 0.3μg/μl stock of SpAGS-mutant GFP mRNAs and 0.25μg/μl stock of 2x-mCherry-EMTB mRNA. White arrowheads indicate vegetal cortical localization of AGS-GFP proteins and ectopic localization of AGS1111 mutant. **C,** % of the embryos with vegetal cortical localization of SpAGS mutants (left) and the ratio of the cortical-to-non-cortical mean intensity (right) in 16~32-cell embryos. Statistical analysis was performed against Full AGS by One-Way ANOVA. **D-F,** Embryos were injected with 0.15μg/μl stock of SpAGS-GFP mRNAs and 0.75mM SpAGS MO. The number of embryos making micromeres (D) and developing to gastrula or pluteus stage (F) were scored and normalized to that of the Full AGS control group. Brightfield images (E) show the representative phenotypes scored in the corresponding graph (F) at 2 dpf. Of note, AGS1111 and AGS2222 mutants caused substantial toxicity, degrading many embryos by 2 dpf and resulting in inconsistent scoring. Thus, we scored embryos reaching the pluteus stage, which revealed delayed development in this analysis. Statistical analysis was performed by One-Way ANOVA. **G,** Design of GFP-SpAGS C-terminal mutant constructs tested in this study. In AGS4444, we replaced all GL motifs with GL4. In AGS-GL1GL2, GL1 is shifted adjacent to GL2. TPR motifs are marked in blue, and GL motifs are in orange. Red boxes show modified GL motifs. **H**¸ Single Z-slice confocal images of sea urchin embryos at 8~16-cell stage showing localization of GFP-SpAGS-GL1GL2 mutant. Embryos were injected with 0.3μg/μl stock of SpAGS-GFP mRNA and 0.25μg/μl stock of 2x-mCherry-EMTB mRNA. The white arrowhead indicates the vegetal cortical localization of GFP-AGS. **I,** % of the embryos with vegetal cortical localization of SpAGS mutants (left) and the ratio of the cortical-to-non-cortical mean intensity (right) in 16~32-cell embryos. Statistical analysis was performed against Full AGS by One-Way ANOVA. **J-K,** Embryos were injected with 0.15μg/μl stock of GFP-SpAGS mRNAs and 0.75mM SpAGS MO. The number of embryos making micromeres (J) and developing to gastrula or pluteus stage (K) were scored and normalized to that of the Full AGS. Statistical analysis was performed by One-Way ANOVA. n indicates the total number of embryos scored. * represents p-value < 0.05, ** p-value < 0.01, *** p-value <0.001, and **** p-value <0.0001. Each experiment was performed at least two independent times. Error bars represent standard error. Scale bars=10μm.

To determine whether the sequence or positioning of GL1 is essential for the SpAGS function, we next made a series of mutants where the GL motifs were interchanged or replaced (Fig. 4A). For instance, AGS1111 has all GL motifs replaced with the sequence of GL1, whereas AGS4234 has the sequence of GL1 replaced with that of GL4. Most embryos that formed micromeres displayed vegetal cortical localization for all mutants except for AGS1111 and AGS2222, which severely inhibited micromere formation (Fig. 4B–C). A small portion (4.14% ± 2.65, n=170) of AGS2222 embryos formed micromeres and always showed vegetal cortical localization, suggesting that AGS localization and micromere formation are closely linked to each other. Additionally, most of all AGS1111 embryos (99.36% ± 0.64, n=182) and of AGS2222 (98.06% ± 1.94, n=170) displayed ectopic cortical localization around the entire embryo (Fig. 4B). We did not observe this phenotype in the Full AGS control nor the other two mutants (AGS2134 and AGS4234).

We quantified the function of these AGS mutants in the endogenous AGS-knockdown background. AGS1111 and AGS2222 mutants failed to restore micromere formation at the 16-cell stage, while AGS4234 and AGS2134 mutants rescued micromere formation similarly to Full AGS (Fig. 4D). Furthermore, Full AGS, AGS2134, and AGS4234 showed comparable development at 2 dpf. In contrast, the AGS1111 and AGS2222 showed disrupted development (Fig. 4E–F). These results suggest that the GL1 sequence is not essential, but its position is vital. In contrast, the sequence of GL3 or GL4 appears to be critical for restricting AGS localization to the vegetal cortex, perhaps by maintaining the autoinhibited form of AGS through their interaction with the TPR domains. AGS1111 and AGS2222 mutants were thus unable to sustain a closed/inactive state, resulting in a constitutively active form all around the cortex.

To test this model further, we made two additional SpAGS mutants, AGS4444 and AGS-GL1GL2 (Fig. 4G). AGS4444 localized properly at the vegetal cortex, whereas AGS-GL1GL2 showed significantly fewer embryos with vegetal cortical localization (Fig. 4H–I). Furthermore, AGS-GL1GL2 showed impaired function in micromere formation and development compared to Full AGS control (Fig. 4J–K). On the other hand, AGS4444 showed no significant difference in the proportion of embryos with micromeres at the 16-cell stage and normal development at 2 dpf compared to the Full AGS control. These results further support the contention that GL3 and GL4 are essential for maintaining the SpAGS in a closed form. Additionally, the position of GL1 is critical for SpAGS localization and function.

### The molecular evolution of the AGS C-terminus facilitates the ACD diversity among AGS orthologs

**Figure 5.**
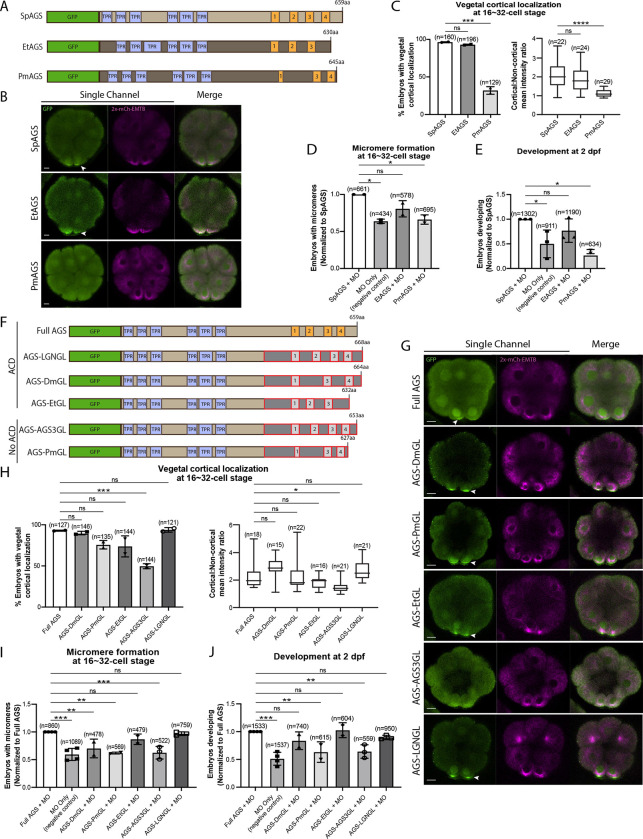
Molecular evolution of AGS C-terminus facilitates micromere formation. **A**, Design of GFP-AGS constructs from three different species tested in this study, namely, *S. purpuratus* (Sp), *E. tribuloides* (Et), and *P. miniata* (Pm). TPR motifs are marked in blue, and GL motifs are in orange. **B¸** Single Z-slice confocal images of sea urchin embryos at 16-cell stage showing localization of each GFP-AGS. Embryos were injected with 0.3μg/μl stock of GFP-AGS mRNAs and 0.25μg/μl stock of 2x-mCherry-EMTB mRNA. The white arrowhead indicates vegetal cortical localization of GFP-AGS. **C,** % of embryos with vegetal cortical localization of GFP-AGS (left) and the ratio of the cortical-to-non-cortical mean intensity (right) in 16~32-cell embryos. Statistical analysis was performed against SpAGS by One-Way ANOVA. **D-E,** Embryos were injected with 0.15μg/μl stock of GFP-AGS mRNAs and 0.75mM SpAGS MO. The number of embryos making micromeres (D) and developing to pluteus stage (E) were scored and normalized to that of the Full AGS. Statistical analysis was performed by One-Way ANOVA. **F**, Design of GFP-AGS C-terminal chimeric mutant constructs tested in this study. TPR motifs are marked in blue, and GL motifs are in orange. The brown section shows the SpAGS portion, and the red and dark grey boxes show the non-sea urchin (non-SpAGS) C-terminal sequence introduced. Protein sequences used include *Drosophila* Pins (Dm), *P. miniata* AGS (Pm), *E. tribuloides* AGS (Et), *H. sapiens* AGS3 (AGS3) and *H. sapiens* LGN (LGN). **G**¸ Single Z-slice confocal images of sea urchin embryos at 8~16-cell stage showing localization of each GFP-AGS. Embryos were injected with 0.3μg/μl stock of GFP-AGS mRNA and 0.25μg/μl stock of 2x-mCherry-EMTB mRNA. The white arrowheads indicate vegetal cortical localization of GFP-AGS. **H,** % of the embryos with vegetal cortical localization of GFP-AGS chimeric mutants (left) and the ratio of the cortical-to-non-cortical mean intensity (right) in 16~32-cell embryos. Statistical analysis was performed against Full AGS by One-Way ANOVA. **I-J,** Embryos were injected with 0.15μg/μl stock of GFP-AGS mRNAs and 0.75mM SpAGS MO. The number of embryos making micromeres (I) and developing to gastrula or pluteus stage (J) were scored and normalized to that of the Full AGS. Statistical analysis was performed by One-Way ANOVA. n indicates the total number of embryos scored. * represents p-value < 0.05, ** p-value < 0.01, *** p-value < 0.001, and **** p-value <0.0001. Each experiment was performed at least two independent times. Error bars represent standard error. Scale bars=10μm.

To understand if/how SpAGS functions uniquely compared to other echinoderm AGS orthologs, we cloned sea star (*P. miniata*; Pm) and pencil urchin (*E. tribuloides*; Et) AGS into the GFP-reporter construct (Fig. 5A) and introduced them into the sea urchin zygotes. EtAGS showed no significant difference compared to the SpAGS control, while PmAGS failed in vegetal cortical localization and micromere formation and function (Fig. 5B–E). Hence, PmAGS is incapable of inducing micromere formation.

Since the N-terminal sequences of SpAGS and PmAGS are almost identical (Fig. S2), we hypothesize that the variable C-terminus made a difference in AGS localization and function at the vegetal cortex. To test this hypothesis, we constructed a series of chimeric SpAGS mutants that replaced its C-terminus with that of other AGS orthologs (Fig. 5F). These AGS orthologs include human LGN, *Drosophila* (Dm) Pins, and EtAGS, which are all involved in ACD (Gonczy, 2008; Schaefer et al., 2000; Wavreil & Yajima, 2020; Zhu et al., 2011a, 2011b) as well as human AGS3 and PmAGS, neither of which is involved in ACD (Saadaoui et al., 2017).

The chimeras of ACD-facilitating orthologs (EtGL, LGNGL, DmGL) showed no significant difference in the vegetal cortical localization and micromere function compared to the SpAGS control (Fig. 5G–J). In contrast, chimeras of no-ACD-facilitators (AGS3GL and PmGL) failed in micromere formation and function. These results suggest that the AGS C-terminus creates ACD diversity by largely reflecting the original function of each ortholog in the host species. Of note, *Drosophila* Pins chimera (DmGL) showed reduced micromere formation (Fig. 5I), which may be due to fewer functional domains with reduced efficacy in the higher-order organism (Wavreil and Yajima, 2020).

Additionally, AGS-PmGL unexpectedly showed cortical localization (Fig. 5G), while PmAGS showed no cortical localization (Fig. 5B). This result suggests that other elements of SpAGS outside of its C-terminus can drive its vegetal cortical localization but not function. Aurora A phosphorylates the linker serine region, enabling Dlg to bind and activate Pins in *Drosophila* (Johnston et al., 2009). To test if this serine is essential for SpAGS localization, we mutated it to alanine (AGS-S389A in Fig. S3A). Compared to the Full AGS control, the mutant AGS-S389A showed reduced vegetal cortical localization (Fig. S3B–C) and function (Fig. S3D–E). Furthermore, we replaced the linker region of PmAGS with that of SpAGS (PmAGS-SpLinker in Fig. S4A–B). However, this mutant did not show any cortical localization nor proper function in ACD (Fig. S4C–F). Therefore, the SpAGS C-terminus is the primary element that drives ACD, while the linker region serves as the secondary element to help cortical localization of AGS.

Lastly, in humans, it is proposed that the interdomain sequence between GL2 and GL3 is important for intramolecular interaction with TPR through phosphorylation, mediating the autoinhibitory state of LGN differently from that of AGS3 (Takayanagi et al., 2019). To test this possibility, we made mutants targeting the residues unique to the AGS3 GL2-GL3 interdomain region (green and red residues in Fig. S5): three serine residues were mutated to alanine, and three other residues (G, N, Y) were replaced with the corresponding residues of LGN. Consistent with our hypothesis, the chimera replaced with the LGN residues (AGS3GL-GL2GL3) gained the proper localization and function, while the chimera with serine alterations (AGS3GL-3S/A) failed to function in ACD (Fig. S3C–E). These results suggest that specific amino acid residues within the GL3 motif are critical, likely mediating interaction with TPR domains and the autoinhibited state of AGS. This result aligns with the earlier results of AGS1111 and AGS2222, which failed in ACD. On the other hand, potential serine phosphorylation between GL2-GL3 motifs appears to be irrelevant to the AGS function.

**Figure 6.**
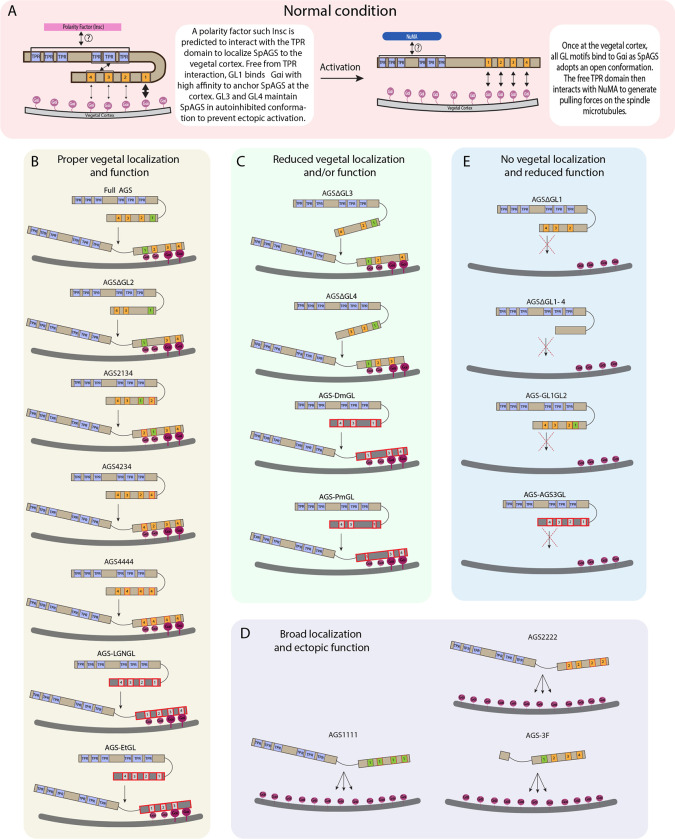
Summary diagrams of SpAGS dissection experiments. **A,** A model for the mechanism of SpAGS localization and function at the vegetal cortex. In a closed conformation, GL1 is critical for SpAGS recruitment and anchoring at the cortex through Gαi binding, while GL3 and GL4 maintain the autoinhibition. The TPR domain is hypothesized to interact with a polarity factor such as Insc to restrict SpAGS localization to the vegetal cortex. Upon Gαi binding, SpAGS adopts an open conformation, allowing all four GLs to bind to Gαi and the TPR domain to interact with NuMA for force generation on the astral microtubules. **B,** A series of mutants that showed normal vegetal localization and functions. The position of GL1 is a more determining factor since mutants with GL1 replaced with other GL sequences localized and functioned properly. **C,** A series of mutants that showed a reduced vegetal localization and/or function. The GL3 and GL4 are necessary to regulate AGS localization and function, likely by mediating its autoinhibitory mechanism through their binding to TPRs. Furthermore, AGS-DmGL and PmGL were categorized in this group due to the reduced number of GL motifs. **D,** A series of mutants that showed broad AGS localization and ectopic function. The TPR domain is critical for restricting AGS localization at the vegetal cortex since its removal spreads the AGS signal around all cortices. The sequences of GL3 and GL4 are also crucial for the SpAGS function. **E,** A series of mutants that showed neither vegetal localization nor function. Removing or displacing GL1 led to significant disturbances in AGS localization and function, suggesting that having a GL motif at this specific position is critical for AGS’ interaction with Gαi and its anchoring to the cortex.

Overall, we conclude that the variable C-terminus of AGS orthologs facilitates ACD diversity. At the same time, the N-terminus and the linker region of AGS appear to help mediate its autoinhibited state, which regulates its cortical localization (summary diagrams in Fig. 6).

### SpAGS is a dominant factor for micromere formation

Since AGS is a part of the conserved ACD machinery, we next sought to understand how dominant SpAGS is for micromere formation. The other conserved ACD factors include Insc, Dlg, NuMA, and Par3 (Fig. 1C). Insc controls cortical localization of Pins and LGN in flies and humans, respectively (Schaefer et al., 2000; Williams et al., 2014; Yu et al., 2000; Culurgioni et al., 2011; Culurgioni et al., 2018). Dlg appears to bind to the phosphorylated linker domain of Pins, which recruits microtubules to the cortex in flies (Johnston et al., 2009; Siegrist & Doe, 2005). NuMA (Mud in *Drosophila*) interacts with LGN/Pins to generate pulling forces on the microtubules in humans and flies. Par3 (Baz in *Drosophila*) is part of the PAR complex with Par6 and aPKC and binds to Insc to help localize LGN/Pins at the cortex (Culurgioni et al., 2011; Parmentier et al., 2000; Schaefer et al., 2000; Schober et al., 1999; Wodarz et al., 2000; Yu et al., 2000).

**Figure 7.**
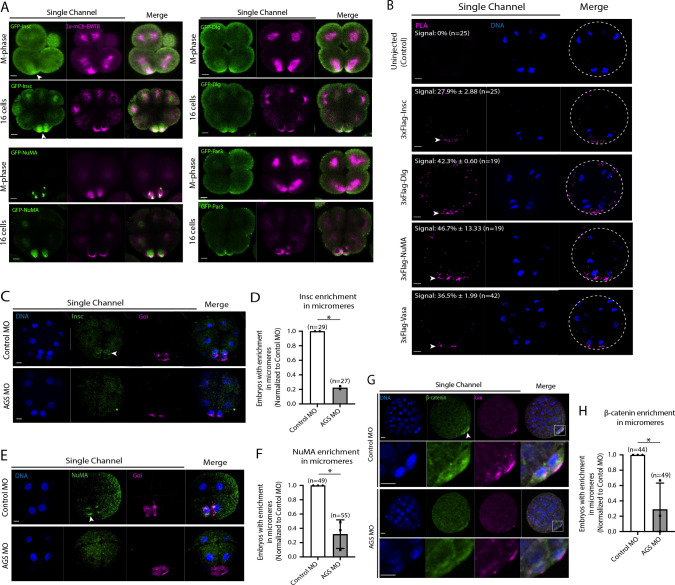
SpAGS is critical for the proper localization of ACD factors and fate determinants. **A,** Single Z-slice confocal imaging was used to focus on the vegetal cortex. Representative images of embryos during the metaphase and at the 16-cell stage show localization of each GFP-ACD factor, SpInsc, SpDlg, SpNuMA, and SpPar3. Embryos were injected with 0.5μg/μl stock of GFP-ACD factor mRNAs and 0.25μg/μl stock of 2x-mCherry-EMTB mRNA. White arrowheads indicate vegetal cortical localization of GFP constructs. Images represent over 80% of the embryos observed (n=30 or larger) per group across at least two independent cycles of experiments. **B,** Single Z-slice confocal images of sea urchin embryos at the 8~16-cell stage showing the signals at the vegetal cortex by PLA assay with Flag and AGS antibodies. Embryos were injected with 0.3–1μg/μl stock of 3xFlag-ACD factor mRNA. White arrowheads indicate the colocalization of AGS and another ACD factor at the vegetal cortex. The average % of the 8-cell and 8~16-cell embryos with the PLA signal across two independent cycles of experiments is indicated in each image. All embryos were scored independently of the angle since it was hard to identify the angle at the 8-cell stage. **C-F,** Representative 2D-projection images of the embryo stained with Insc (C), NuMA (E), and β-catenin (G) antibodies (green) by immunofluorescence. Embryos were stained with Gɑi antibody (magenta) and Hoechst dye (blue) as well. Z-stack images were taken at 1μm intervals to cover a layer of the embryo. White arrowheads indicate the signal in micromeres. Embryos were injected with 0.75mM Control MO or 0.75mM SpAGS MO. The number of embryos showing the localization of Insc (D), NuMA (F), and β-catenin (H) in micromeres were scored and normalized to that of the Control MO. Statistical analysis was performed by *t*-test. n indicates the total number of embryos scored. * represents p-value<0.05. Each experiment was performed at least two independent times. Error bars represent standard error. Scale bars=10μm.

We cloned the sea urchin orthologs of these ACD factors and tagged each ORF with a GFP reporter. All ACD factors showed precise vegetal cortical localization during or upon micromere formation by GFP live imaging or immunofluorescence (Fig. 7A and S6). Furthermore, we tested for physical interaction by performing the proximity ligation assay (PLA) for AGS and ACD factors (Insc, NuMA, Dlg). The results suggest these factors physically interact with AGS at the vegetal cortex (Fig. 7B). Hence, the core ACD machinery is present at the vegetal cortex and interacts with AGS. We also observed AGS interacting with a fate determinant, Vasa, that is known to be enriched in micromeres at the vegetal cortex (Fig. 7B) (Voronina et al., 2008). These results indicate that AGS may recruit both ACD factors and fate determinants to the vegetal cortex, directly facilitating rapid lineage segregation of micromeres.

**Figure 8.**
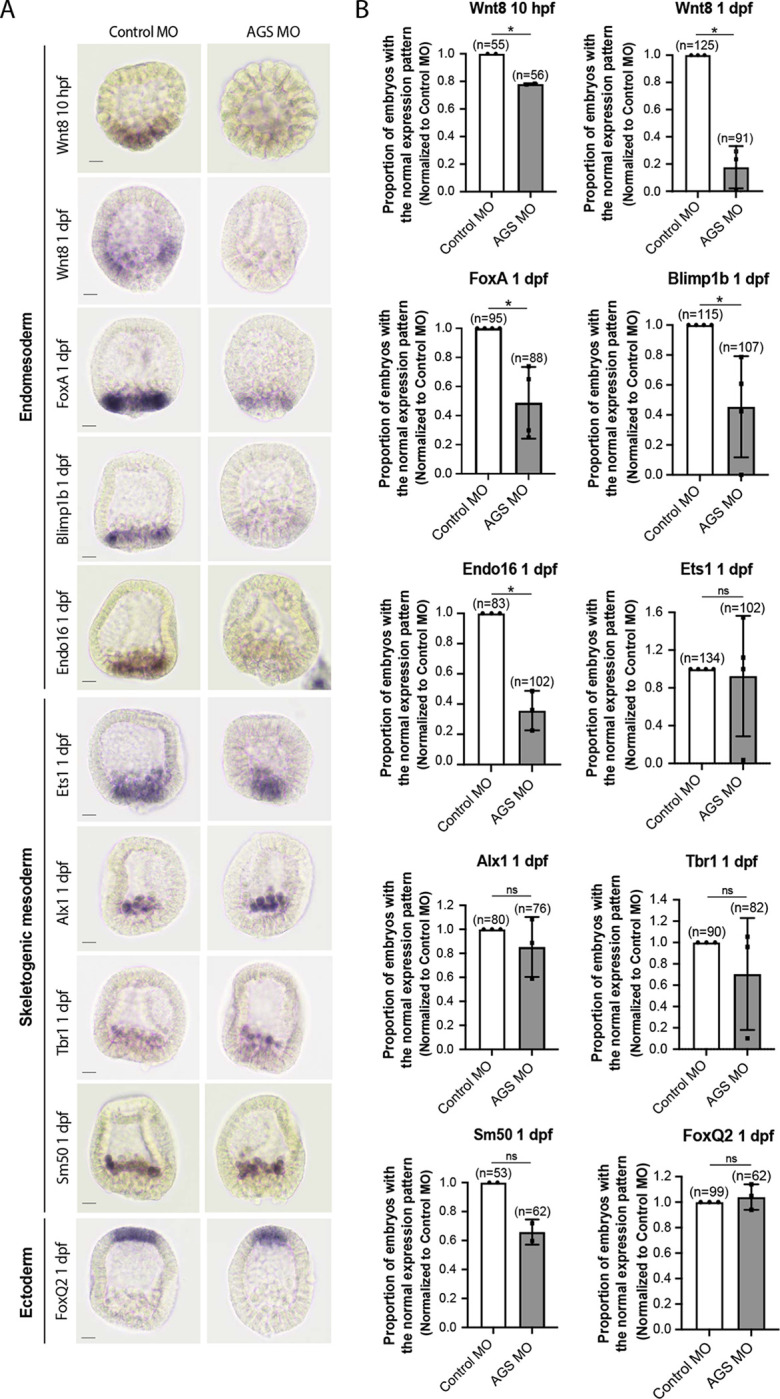
SpAGS is critical for the downstream gene expressions facilitated by micromere signaling. Embryos were injected with 0.75mM Control MO or 0.75mM SpAGS MO. Brightfield images (A) show the representative ISH staining for each cell lineage marker scored in the corresponding graph (B). The number of embryos showing the normal signal patterns of each marker gene was scored and normalized to that of the Control MO. Statistical analysis was performed by *t*-test. n indicates the total number of embryos scored. * represents p-value < 0.05. Each experiment was performed at least two independent times. Error bars represent standard error. Scale bars=20μm.

Consistent with this observation, SpAGS knockdown reduced the signal enrichment of ACD factors and another fate determinant of micromeres, β-catenin (Logan et al., 1999) (Fig. 7C–H). Furthermore, in our previous study (Poon et al., 2019), we identified that SpAGS recruits the spindle poles to every cortex when overexpressed (Fig. S7A, arrows). Similarly, SpAGS at least partially recruits its partner proteins to the ectopic cortical region, which we never observed in the control group (Fig. S7B–C, arrows). These results support the idea that SpAGS directly recruits the molecules essential for micromere lineage segregation. Indeed, *in situ hybridization* (ISH) analysis suggests that the downstream genes regulated by micromere signaling, such as endomesoderm marker genes (*wnt8*, *foxa*, *blimp1b,* and *endo16*), decreased their expression territories in the AGS-knockdown embryos (Fig. 8). In contrast, ectoderm (*foxq2*) and skeletogenic mesoderm (*ets1, alx1, tbr1,* and *sm50)* marker genes showed no significant change in their expressions by AGS knockdown. Overall, these results suggest that SpAGS directly recruits multiple ACD factors and fate determinants necessary for micromere formation and function as an organizer, facilitating the downstream gene expressions necessary for endomesoderm specification.

### AGS serves as a variable factor in the conserved ACD machinery

**Figure 9.**
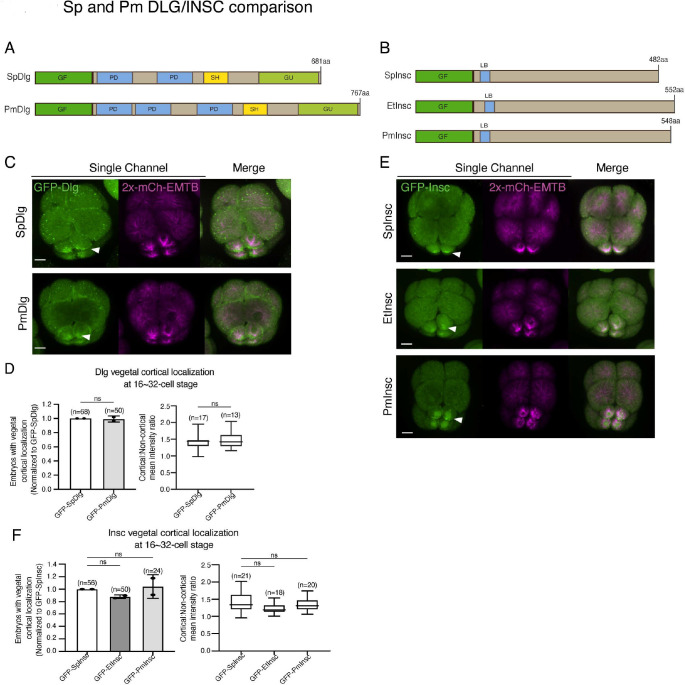
Dlg and Insc are not the variable factors of the ACD machinery in evolution. **A-B**, The design of GFP-Dlg (A) and GFP-Insc (B) constructs that were tested in this study. Of note, EtDlg was unavailable in the database due to the limited genomic information available for this species. **C-F**, Representative 2D-projection images of sea urchin embryos at 8~16-cell stage showing localization of each echinoderm GFP-Dlg (C) and GFP-Insc (E). Z-stack images were taken at 1μm intervals. Embryos were injected with 0.5μg/μl stock of GFP-Dlg or GFP-Insc mRNAs and 0.25μg/μl stock of 2x-mCherry-EMTB mRNA. White arrowheads indicate vegetal cortical localization of GFP constructs. The number of embryos with vegetal cortical localization of GFP-Dlg (D) and GFP-Insc (F) in 16~32-cell embryos was scored and normalized to that of the GFP-SpDlg or GFP-SpInsc (left graph). Right graph shows the ratio of the cortical-to-non-cortical mean intensity. Statistical analysis was performed by *t*-test or One-Way ANOVA. n indicates the total number of embryos scored. Each experiment was performed at least two independent times. Error bars represent standard error. Scale bars=10μm.

AGS shows a variable C-terminal domain and appears to be a primary factor for facilitating ACD diversity. However, is AGS the only variable factor among the ACD machinery? To test this question, we cloned and injected orthologs of other ACD factors, such as Insc and Dlg, from pencil urchins (Et) or sea stars (Pm) into sea urchins. Both Insc and Dlg showed relatively conserved functional domains among three echinoderms with an extra PDZ domain present in PmDlg (Fig. 9A–B; S8–9). Importantly, these Pm and Et ACD factors showed cortical localization at the vegetal cortex in the sea urchin embryo (Fig. 9C–F). These results are in stark contrast to the earlier results of Pm/Et AGS, which showed varied localization and function in ACD. Therefore, Insc and Dlg might not be the significant variable factors.

**Figure 10.**
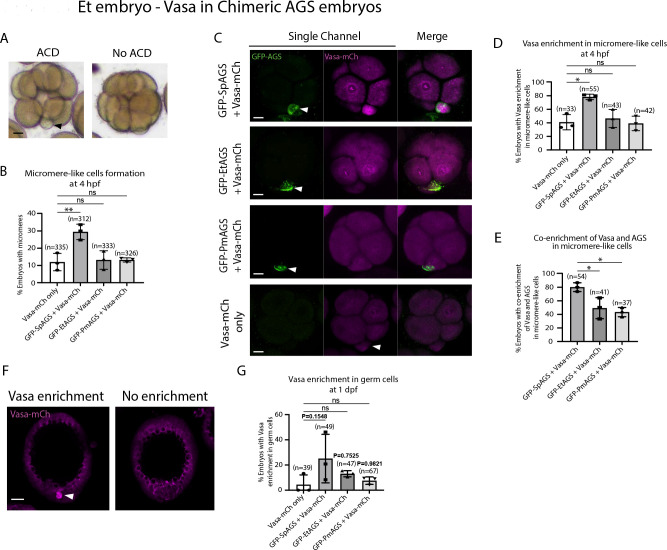
SpAGS but not EtAGS and PmAGS can induce a functional ACD in pencil urchin (Et) embryos. **A,** Representative brightfield images of Et embryos with or without micromere-like cells. Black arrowhead indicates micromere-like cells. **B,** Et embryos were injected with 0.3μg/μl stock of GFP-AGS mRNAs and 1μg/μl stock of Vasa-mCherry mRNA. The number of embryos making micromere-like cells was scored and normalized to that of the Vasa-mCherry only. Statistical analysis was performed against Vasa-mCh only by One-Way ANOVA. **C,** Representative 2D-projection images of the injected Et embryos. Z-stack images were taken at 1μm intervals to cover a layer of the embryo. White arrowheads indicate micromere-like cells. Scale bars=10μm. **D,** The number of embryos showing Vasa enrichment in the micromere-like cells was scored and normalized to that of the Vasa-mCherry only. Only the embryos that formed micromere-like cells were scored. Statistical analysis was performed against Vasa-mCh only by One-Way ANOVA. **E,** % of total embryos showing co-enrichment of Vasa and AGS in the micromere-like cells. Statistical analysis was performed against GFP-SpAGS by One-Way ANOVA. **F-G,** Representative 2D-projection images of Et embryos at 1 dpf. White arrowhead indicates Vasa enrichment in germ cells. Z-stack images were taken at 1μm intervals. % of total embryos showing Vasa enrichment in germ cells at 1 dpf was scored. Et embryos were injected with 0.3μg/μl stock of GFP-AGS mRNAs. Statistical analysis was performed against Vasa-mCh only by One-Way ANOVA. n indicates the total number of embryos scored. * represents p-value < 0.05, and ** p-value < 0.01. Each experiment was performed at least three independent times. Error bars represent standard error. Scale bars=20μm.

To determine how dominantly SpAGS facilitates ACD diversity, we introduced SpAGS, EtAGS, or PmAGS into the pencil urchin, an ancestral type of sea urchin, and compared their function. We co-introduced Vasa-mCherry to identify the development of the germline, which is one of the micromere descendants. Pencil urchin embryos typically form 0–4 micromere-like cells randomly (Fig. 10A). Notably, only SpAGS injection increased the formation of micromere-like cells in the resultant pencil urchin embryos. In contrast, EtAGS and PmAGS showed no significant difference from the negative control (Vasa-mCherry only, Fig. 10B). This result suggests that SpAGS increases the frequency of micromere-like cell formation in pencil urchin embryos.

Sea urchin embryos show Vasa enrichment in micromeres at the 16-cell stage. In contrast, pencil urchin embryos show such enrichment later in the larval stage (3–4 dpf), which is more similar to the timing of the germline segregation in sea star embryos (Juliano & Wessel, 2009). We observed that SpAGS increased the Vasa signal enrichment in micromere-like cells compared to the control (Vasa-mCherry only) at the 16-cell stage. On the other hand, other AGS orthologs showed no significant difference from the control (Fig. 10C–D). Nearly 80% (80.12% ± 3.75) of the SpAGS-introduced embryos showed co-enrichment of AGS and Vasa in micromere-like cells, while the EtAGS and PmAGS groups showed only 49.2% ± 8.94 and 43.37% ± 3.94 enrichment, respectively (Fig. 10E). Consistently, the SpAGS group showed the earlier segregation of Vasa-positive cells similar to sea urchin embryos at 1 dpf (Fig. 10F–G), potentially accelerating the lineage segregation of the pencil urchin embryo.

## Discussion

The introduction of ACD in early embryogenesis of the sea urchin led to the formation of a new cell type, micromeres, with a critical organizer function. In the sea urchin, SpAGS is essential for micromere formation, while other echinoderm embryos show no cortical AGS localization (Poon et al., 2019). This study demonstrates that the GL1 motif of SpAGS is key for its vegetal cortical localization and function in micromere formation. Importantly, this unique role of the GL1 motif appears to be conserved across organisms. In *Drosophila* Pins and humans LGN, GL1 is free from TPR binding, making it essential for the recruitment of Pins/LGN to the cortex (Nipper et al., 2007; Takayanagi et al., 2019). Thus, the evolutionary introduction of the GL1 motif into SpAGS likely increased recruitment affinity to the vegetal cortex, inducing ACD in the sea urchin embryo.

The GL1 deletion significantly disrupted micromere formation, while its replacement with other GL motifs had no effect. Therefore, the GL1 position rather than the sequence is essential for SpAGS function in ACD regulation. In contrast, GL3 and GL4 sequences are crucial for SpAGS activity, which also appears to be conserved across organisms. In *Drosophila* Pins and human LGN, GL2–3 and GL3–4 sequences, respectively, are essential for their intramolecular interactions with TPR motifs, which control Pins/LGN’s autoinhibited conformation (Nipper et al., 2007; Pan et al., 2013; Smith & Prehoda, 2011; Takayanagi et al., 2019). In a closed conformation, Pins/LGN are unable to bind to Mud/NuMA. Therefore, Gαi binding to GL1 relieves the autoinhibition (Du & Macara, 2004; Nipper et al., 2007; Takayanagi et al., 2019; Pan et al., 2013). Indeed, the TPR4–6 motifs are necessary to restrict SpAGS localization to the vegetal cortex, suggesting their interactions with GL motifs to maintain autoinhibition.

While the role of AGS protein in spindle orientation has been established in several model organisms, it was unknown if or how far AGS could regulate the fate determinants to facilitate ACD diversity. In this study, we learned that SpAGS is essential for the recruitment of ACD factors, such as Insc and NuMA, and fate determinants, such as Vasa and β-catenin, to micromeres. Notably, in pencil urchin embryos, SpAGS recruited Vasa protein into micromeres, suggesting SpAGS may be sufficient to recruit necessary fate determinants to create cell lineage segregation in another species. Although such a lineage segregation of micromeres may be mediated solely by ACD, their function as organizers might require additional changes in the developmental program of the entire embryo. For example, sea urchin embryos have a robust hyaline layer to keep blastomeres together, which presumably increases the cell-cell interaction and may also enhance cell signaling during early embryogenesis. In contrast, a hyaline layer is not or little present in sea star or pencil urchin embryos, respectively. At present, we do not know what developmental changes are upstream or downstream of micromere formation during sea urchin diversification. It will be important to identify in the future how far SpAGS impacts the developmental program other than inducing ACD and what other developmental elements play critical roles in establishing micromeres as a new cell lineage and organizers during sea urchin diversification.

Overall, we conclude that the GL1 motif unique to sea urchin AGS orthologs is critical for SpAGS function in micromere formation. Since the unique role of the GL1 motif appears to be conserved across organisms, including *Drosophila* and humans, it is possible that the GL1 motif was once lost in the echinoderm common ancestor and recovered during sea urchin diversification. The recovery of this GL1 motif also resumed the interaction between SpAGS and other ACD machinery, such as NuMA, Insc, and Dlg, at the cortex in a similar manner to its orthologs Pins and LGN in other phyla, resulting in the controlled ACD and further interactions with fate determinants to form a new cell type in the sea urchin embryo. Therefore, unlike random unequal cell divisions that do not alter cell fates, AGS-mediated cell divisions appear to be highly organized and may be programmed to cause cell fate changes. Considering great variations within the C-terminus of AGS orthologs and their immediate impact on micromere formation, we propose that AGS is a variable factor in facilitating ACD diversity among echinoderm embryos, contributing to developmental diversity within a phylum. Future studies in other taxa are awaited to prove this concept further.

## Methods

### Animals and Echinoderm embryos

*Strongylocentrotus purpuratus* (sea urchins) were collected from the ocean by Pat Leahy, Kerchoff Marine Laboratories, California Institute of Technology, or Josh Ross, South Coast Bio-Marine LLC. Long Beach, California, USA, and kept in an aquarium cooled to 16°C. *Eucidaris tribuloides* (pencil urchins) were collected from the ocean by KP Aquatics LLC. in Tavernier, Florida, and maintained in the aquarium at room temperature. Gametes were acquired via 0.5M KCl injections. Eggs were collected in seawater (SW), and sperm was collected dry. For injection, eggs were de-jellied using pH 4.0 SW and placed in a plate coated with protamine sulfate. These eggs were then fertilized and injected in the presence of 1mM 3-amino triazole (Sigma, St. Louis, MO, USA) to prevent crosslinking of fertilization envelopes, and embryos were cultured in SW at 16°C. For protein collection for immunoprecipitation, eggs were fertilized in 1mM 3-amino triazole. Fertilization envelopes were removed by pipetting, and fertilized eggs were placed in a plate coated with the fetal bovine serum to prevent eggs from sticking to the plate.

### Plasmid construction

All constructs were prepared in pSP64 or pCS2 vectors, which were optimized for *in vitro* transcription. SpAGS was previously identified in the sea urchin (Voronina & Wessel, 2006) and SpAGS-GFP was constructed by PCR amplification of the SpAGS ORF, then subcloned into the pSp6 β-globin UTR plasmid between the Xenopus β-globin 5′ and 3′ UTRs as described in Poon et al. (2019) (Fig. S1A). To remove GL1 (473aa DNFFEALSRFQSNRMDEQRCSF 495aa) from SpAGS-GFP, the internal Bbvc1 (458a) and Bsm1 (532aa) sites were used to remove the sequence, including GL1, and the corresponding sequence lacking only GL1 (gBlock, IDT, Iowa, USA) was fused back using In-Fusion HD Cloning kit according to manufacturer’s protocol (#639648, Clontech, USA) (Fig. S1B). The other C-terminal deletion constructs were created following the same method using the internal BbvC1 (458aa) and vector Apa1 sites to remove the original sequence and replace it with each DNA fragment (gBlock, IDT) with the desired sequence. The N-terminal deletion constructs were constructed by removing the entire AGS ORF from the SpAGS-GFP plasmid using the vector Bgl2 and Apa1 sites, then replacing it with a custom DNA fragment (gBlock, IDT), each with the appropriate deletion. The ORF of Insc, Dlg, NuMA, and Par3 was PCR amplified and subcloned into the pSP64-GFP/mCherry vector. The 3xFlag DNA fragment (gBlock, IDT) was inserted into pSP64-GFP-SpInsc/SpDlg/NuMA and pSP64-Vasa-GFP for PLA analysis. pCS2–2x-mCherry-EMTB (#26742 Addgene) (Miller & Bement, 2009) was obtained from Addgene. pSP64-Vasa-mCherry was previously constructed in Uchida and Yajima (2018). pSP64-Vasa-GFP and pSP64-AGS-mCherrywere previously built and used (Fernandez-Nicolas et al., 2022; Poon et al., 2019; Yajima & Wessel, 2011, 2015)

### mRNA injection and microscopy

Constructs were linearized with the appropriate restriction enzymes overnight (Not1 for pCS2–2x-mCherry-EMTB constructs, SmaI, SalI, or EcoRI for all pSP64 constructs), then transcribed *in vitro* with mMESSAGE mMACHINE SP6 Transcription Kit (#AM1340, Thermo Fisher Scientific) which involved a four h incubation at 37°C, followed by a DNaseI treatment and LiCl precipitation overnight at −20°C. Sea urchin embryos were injected at the 1-cell stage with 0.15–1μg/μl of each mRNA as individually indicated. A morpholino antisense oligonucleotide (MO) that explicitly blocks the translation of SpAGS was previously designed and used in Poon et al. (2019). The SpAGS MO sequence is listed below (Supplementary Table 1). For knockdown experiments, embryos were co-injected with 0.75mM MO with or without 0.15μg/μl of SpAGS-GFP mRNA. Embryos were imaged using the Nikon CSU-W1 Spinning disk laser microscope.

### Insc antibody production and validation

Three affinity-purified rabbit antibodies against SpInsc were made by Genscript (Piscataway, NJ). Antibody#1 showed the most specific vegetal cortex signal by immunofluorescence (Fig. S6A). This antibody detected multiple bands yet still displayed the primary band at the expected size (53 kDa) by immunoblot (Fig S6B). The competition assay with SpInsc-peptide removed all bands except for the band at 15kDa (Fig. S6C). Thus, the larger bands detected by this antibody may be the complexes of Insc proteins since Insc is known to form dimers and hexamers with LGN (Culurgioni et al., 2018).

### Immunoblotting

Samples were run on a 10% Tris-glycine polyacrylamide gel (Invitrogen, Carlsbad, CA) before transfer on a nitrocellulose membrane for immunoblotting with Insc antibodies used at 1:2000 dilution with 1.5% BSA, or Actin (#3700S, Cell Signaling Technology) antibody at 1:5000 dilution with 0% BSA, followed by treatment with HRP-conjugated anti-Protein A (ab7245, Abcam) for Insc or HRP-conjugated anti-mouse (#7076, Cell Signaling Technology) secondary antibody for Actin at 1:2000. The reacted proteins were detected by incubating the membranes in the chemiluminescence solution (luminol, coumaric acid, hydrogen peroxide, Tris pH 6.8) and imaged by the ChemiDoc Gel Imaging System (BioRad, USA).

### Immunofluorescence

The final concentrations of primary antibodies were anti-SpInsc at 1:200, anti-SpAGS (Poon et al., 2019) and anti-βcatenin (Yazaki et al., 2015) at 1:300, anti-SpNuMA (Poon et al., 2019) at 1:500, and anti-Gαi (#sc-56536, Santa Cruz Biotech) at 1:30. The secondary antibodies were used at a dilution of 1:300 Alexa 488-conjugated goat anti-rabbit (#4412, Cell Signaling Technology) or Alexa 555-conjugated goat anti-mouse (#4409, Cell Signaling Technology). Hoechst dye (#62249, Thermo Fisher Scientific) at 1:1000 (10 mg/mL stock) was used to visualize DNA. Embryos of the desired developmental stage were fixed with 90% cold methanol for more than 1 hour at −20°C, washed with 1X PBS, and incubated with the primary antibody overnight at 4°C, followed by 10 washes with 1X PBS, then incubated with the secondary antibody at room temperature for 3 h. The secondary antibody was washed 10 times with 1X PBS and Hoechst treatment for 15 min. Samples were plated onto slides. All fluorescent images were taken under the Nikon CSU-W1 Spinning disk laser microscope.

### Proximity ligation assay (PLA)

Embryos at the 8~16-cell stage were fixed with 90% cold methanol for over 1 hour at −20 °C, washed with 1X PBS, and treated with 0.05% Triton-X for 15 min. PLA was processed following a manufacturer’s protocol (#DUO92101, Millipore-Sigma). The concentration of primary antibodies was anti-SpAGS (Voronina & Wessel, 2006) at 1:300 and anti-Flag (#F1804, Millipore-Sigma) at 1:100. Embryos were taken images under the Nikon CSU-W1 Spinning disk laser microscope.

### In situ hybridization (ISH)

The embryos were fixed using 4% paraformaldehyde at the ideal stage. Fixed embryos were washed with MOPS buffer and stored in 70% EtOH at −20 °C until needed. In situ hybridization was performed as previously described (Minokawa et al., 2004; Perillo et al., 2021). Sequences used to make antisense probes were PCR amplified from 1 dpf embryonic cDNA of either sea urchin or pencil urchin (Erkenbrack, 2016; Erkenbrack & Davidson, 2015; Erkenbrack et al., 2018) and cloned into TOPO vector (#45–124-5, Thermo Fisher Scientific) (Supplementary Table 1). The TOPO plasmids were linearized using BamHI or HindIII (T7 transcription) and NotI or XhoI (SP6 transcription) for subsequent *in vitro* transcription using either SP6 or T7 MEGAscript Transcription kit (#AM1330 or AM1333, Thermo Fisher Scientific) with DIG RNA labeling mix (#11277073910, Roche; Indianapolis, IN).

### Data analysis

All quantitative data were analyzed using GraphPad Prism 8.3.1 software. Each experiment was repeated at least two independent times. Statistical significance was determined by a *t*-test or One-Way ANOVA. p values less than 0.05. * indicates p-value < 0.05, ** p-value < 0.01, *** p-value < 0.001 and **** p-value < 0.0001.

### Blast and motif analysis

All echinoderm sequences were obtained from Echinobase.org. Protein sequence alignment and molecular phylogenetic tree were constructed using *Clustal Omega* and *CIPRES Science Gateway V. 3.3*. Protein structural motif analysis was performed through the NCBI blast search of the database CDD v3.17 with the value threshold of 0.02. The GoLoco (GL) motif found in the C-terminal of AGS-family proteins is defined by a conserved core of 19 amino acids except for the *C. elegans,* where the single GL motif is 18 amino acids long (Willard et al., 2004). In Fig. 1B, some GL or TPR motifs were considered partial as they are predicted to be less than 18 amino acids long, or a few amino acids are altered in the motif, respectively. Each GL motif was numbered according to sequence similarity to that of *S. purpuratus* AGS GL motifs.

## Supplementary Material

Supplement 1

## Highlights

- Evolutionary modifications of GoLoco motifs are critical for AGS function in micromere formation in the sea urchin embryo.
- The chimeric AGS, which contains the C-terminus of AGS orthologs from various organisms, suggests that human LGN, pencil urchin AGS, and *Drosophila* Pins compensate for the activity of sea urchin AGS.
- Sea urchin AGS regulates the localization of the conserved asymmetric cell division (ACD) machinery members at the vegetal cortex.
- SpAGS is a variable factor facilitating ACD diversity during species diversification.
