# Supplementary material for: The evolutionary modifications of a GoLoco motif in the AGS protein facilitate micromere formation in the sea urchin embryo
Source: bioRxiv. 2024 Oct 15:2024.06.30.601440. Originally published 2024 Jul 2. Preprint. [Version 2] doi: 10.1101/2024.06.30.601440 (PMC11244941; doi:10.1101/2024.06.30.601440)
Supplement: Supplement 1 [file NIHPP2024.06.30.601440v2-supplement-1.pdf]

bioRxiv preprint doi: https://doi.org/10.1101/2024.06.30.601440; this version posted October 15, 2024. The copyright holder for this preprint (which was not certified by peer review) is the author/funder, who has granted bioRxiv a license to display the preprint in perpetuity. It is made available under aCC-BY 4.0 International license.

Supplementary Figures and Legends

Supplemental Figure S1

A

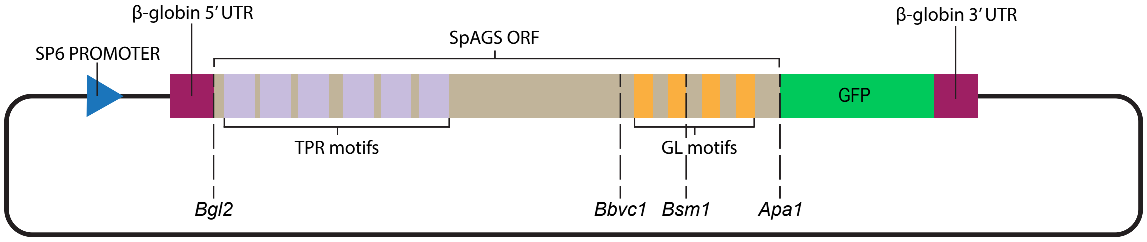

B

S. purpuratus AGS Protein Sequence

TPR1 / TPR2
MQSEASCMELALEGERLCKAGNCREGVRYLEAAVEVGTDDLKTLSAIYSQLGNAYFYLEDYGKALTYHKHDL

TPR3 / 117aa, before here removed in 1F
TLATSIGDRLGEAKASGNIGNTLKVLGKFDEAICCCQRHLDISRELSEKVGEGRALYNLGNVYHAKGKHAGRS

TPR4
GHQDPGDFPQEVTDCLKQAIQFYEANLAIVRELGDRAAQGRACGNLGNTHYLLGNFETAIQFHTERLAIAKE

222aa, before here removed in 2F / TPR5 / TPR6
FGDKAAERRAYSNLGNACVFMVQFEVAAKYYKKSLHIARQLGELAMEAQACYSLGNTYTLLREYEKAVEYHS

301aa, before here removed in 3F
RHMEIAQQLNDRVGEGRACWSLGNAHTSLGNHEKALHYATLHLQISREVGDRTGEVTAKMNLQDLQTLFG

ISTADLSEVSTTVQTPLQESKGARPRRRRSMENLELVAMTPEKKTEIVQVPKRKIRPGSKLKLKNGGESKDKQS

BbvC1 RE site / GL1 (removed in Δ1)
HKDSKESVSSAKNSVPAPSKAKSQTSARKGGEPIQMLDDADNFFEALSRFQSNRMDEQRCSFGRLQKKLAD

GL2 (removed in Δ2) / Bsm1 RE site / GL3 (removed in Δ3)
EEGNGLPEKEELLDEIAKLQGSRLNEQRAFSVKRLPGLPGLRANEDVVGKLLAKGERAEPDDDFFEMIIRCQG

GL4 (removed in Δ4)
ARIEDQRSTLPIQAPAPTVPDEDFFSLIQRIQSKRIEEQRSIAPWEKGSGTSCVCFYDYDTSRTEGTACCVYSRP

659aa
VQESHL

Supplemental Figure 1. **Dissection of SpAGS motifs. A**, The construct design of SpAGS-GFP, including the restriction enzyme sites used to prepare SpAGS mutants. **B**, The protein sequence of SpAGS. Predicted domains are labeled based on NCBI blast results, and the sequence portions deleted for each N-terminal construct are marked. The sequences for each GL motif used for deletion or swapping are indicated in orange. The internal restriction enzyme sites for *BbvCI* and *BsmI* are shown in green.

bioRxiv preprint doi: https://doi.org/10.1101/2024.06.30.601440; this version posted October 15, 2024. The copyright holder for this preprint (which was not certified by peer review) is the author/funder, who has granted bioRxiv a license to display the preprint in perpetuity. It is made available under aCC-BY 4.0 International license.

Supplemental Figure 2   Echinoderm AGS sequence alignment

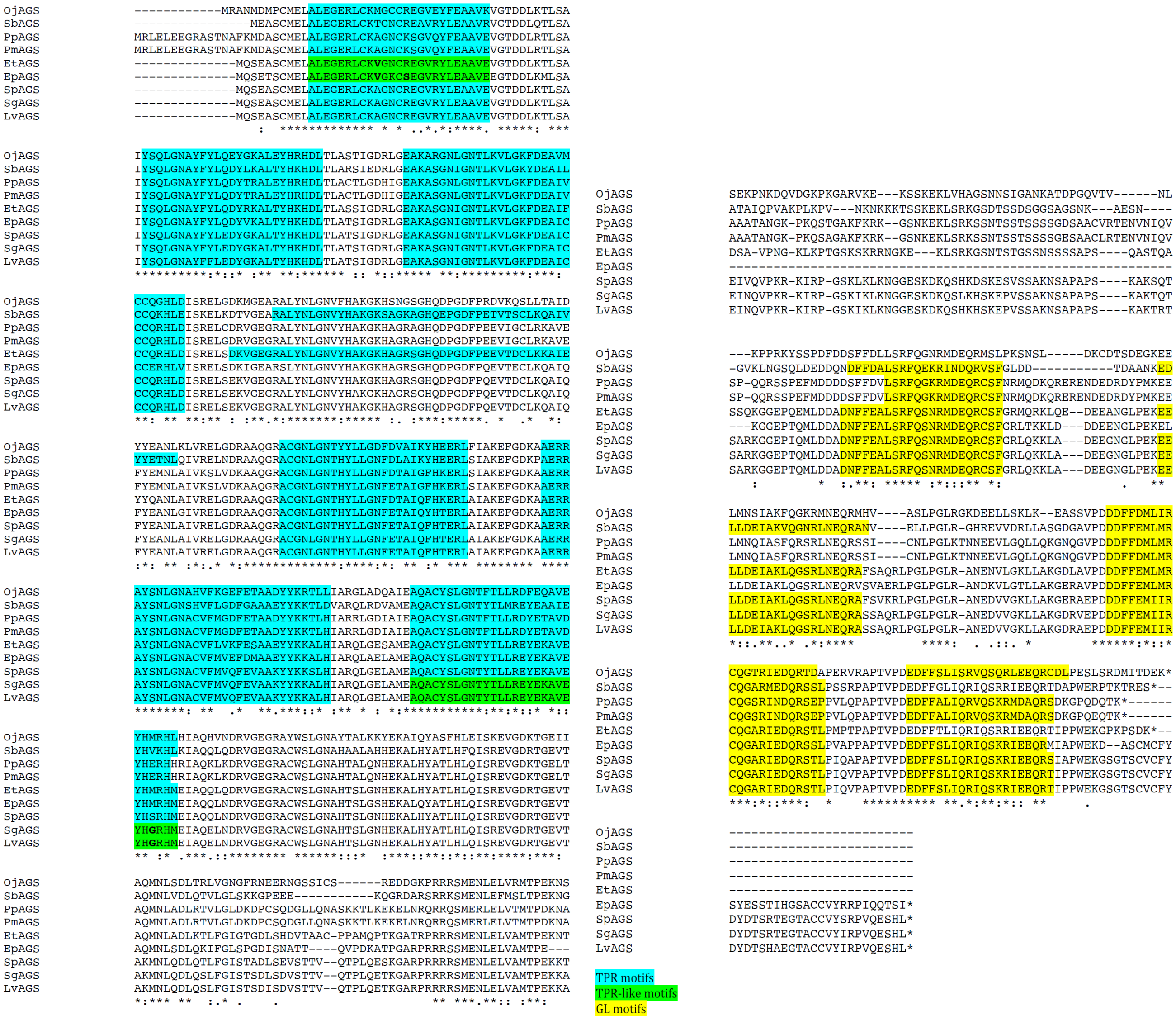

Supplemental Figure 2. Echinoderm AGS sequence alignment. All AGS are similar in the N-terminus with the predicted conserved TPR motifs (blue) but are highly variable in the C-terminus with the predicted GL motifs (yellow).

bioRxiv preprint doi: https://doi.org/10.1101/2024.06.30.601440; this version posted October 15, 2024. The copyright holder for this preprint (which was not certified by peer review) is the author/funder, who has granted bioRxiv a license to display the preprint in perpetuity. It is made available under aCC-BY 4.0 International license.

Supplemental Figure 3

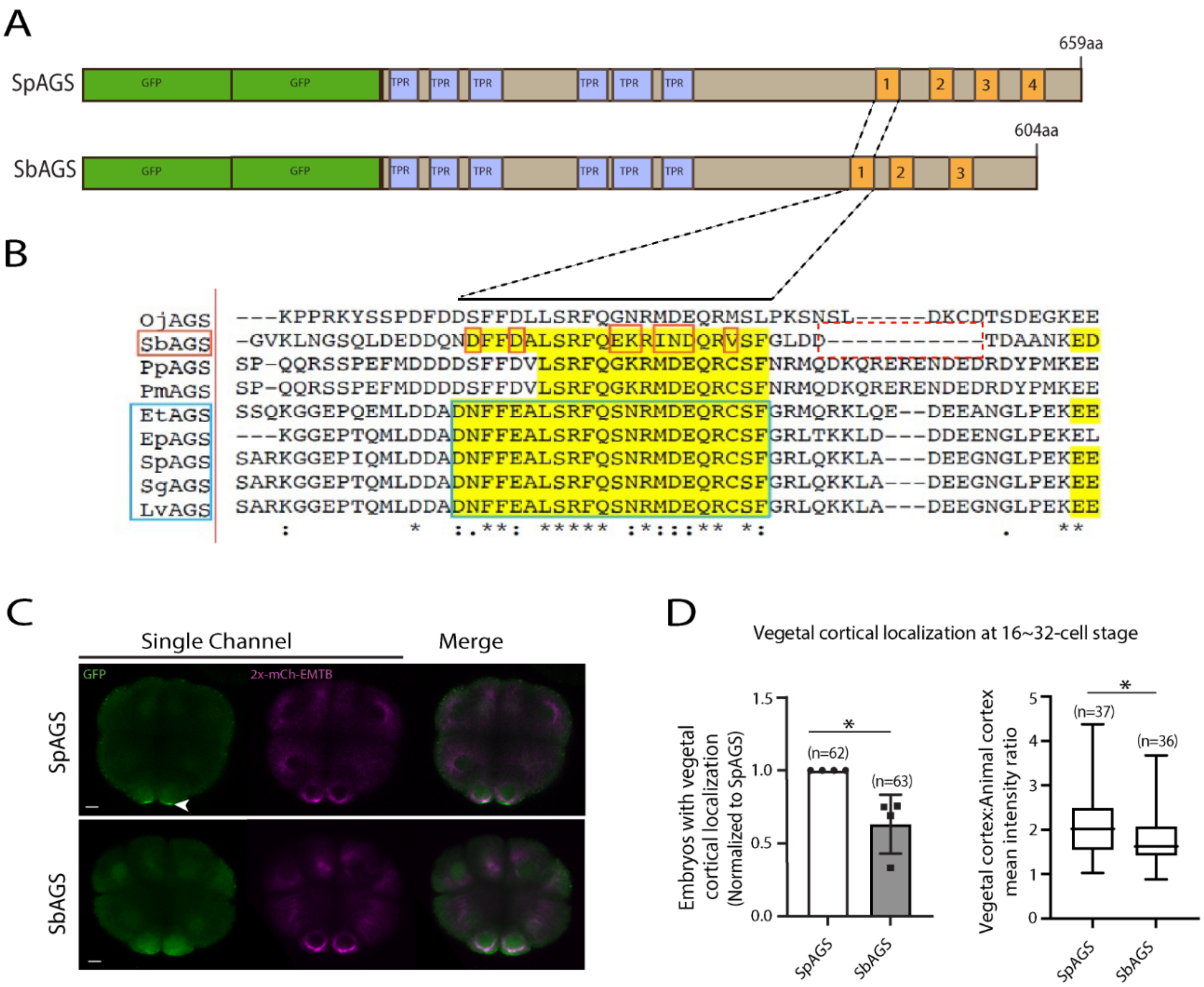

Supplemental Figure 3. SbAGS does not fully localize at the vegetal cortex. A, Design of 2XGFP-AGS constructs that contain AGS orthologs from two different species tested in this study, namely, *S. purpuratus* (Sp; sea urchin), and *S. briareus* (Sb; sea cucumber). TPR motifs are marked in blue, and GL motifs are in orange. **B¸** Alignment of GL1 motif sequences among echinoderms. **C,** Single Z-slice confocal images of sea urchin (Sp) or sea cucumber (Sb) embryos at 16-cell stage showing localization of 2x-GFP-AGS. Embryos were injected with 0.2-0.3µg/µl stock of GFP-AGS mRNA and 0.25µg/µl stock of 2x-mCherry-EMTB mRNA. The white arrowhead indicates vegetal cortical localization of GFP-AGS. **D,** Left graph, the number of embryos with vegetal cortical localization of 2x-GFP-AGS in 16~32-cell embryos was scored and normalized to that of the control group (SpAGS). Right graph, the ratio of the vegetal cortex-to-animal cortex mean intensity. Statistical analysis was performed against the control (SpAGS) by *t*-test. n indicates the total number of embryos scored. * represents p-value < 0.05. Each experiment was performed at least two independent times. Error bars represent standard error. Scale bars=10µm.

bioRxiv preprint doi: https://doi.org/10.1101/2024.06.30.601440; this version posted October 15, 2024. The copyright holder for this preprint (which was not certified by peer review) is the author/funder, who has granted bioRxiv a license to display the preprint in perpetuity. It is made available under aCC-BY 4.0 International license.

Supplemental Figure 4 SpAGS, EtAGS and PmAGS linker domain sequence alignment

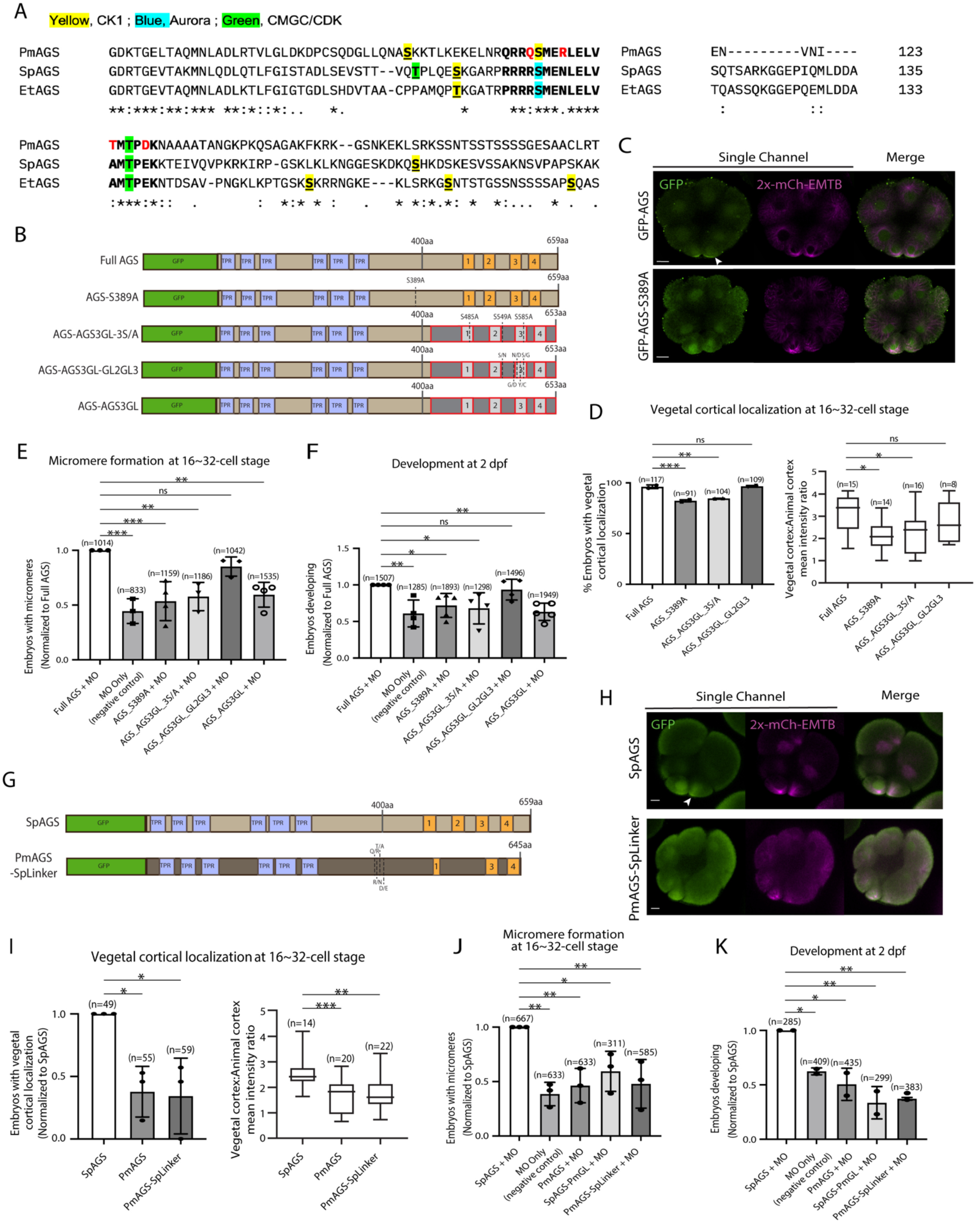

bioRxiv preprint doi: https://doi.org/10.1101/2024.06.30.601440; this version posted October 15, 2024. The copyright holder for this preprint (which was not certified by peer review) is the author/funder, who has granted bioRxiv a license to display the preprint in perpetuity. It is made available under aCC-BY 4.0 International license.

Supplemental Figure 4. The linker domain and GL2-GL3 regions are important for AGS localization and function. **A**, Alignment of linker domain between echinoderms including sea urchin (SpAGS), pencil urchin (EtAGS), and sea star (PmAGS). Bold letters represent the conserved core linker domain. The yellow, blue, or green highlights indicate the CK1, Aurora, or CMGC/CDK phosphorylation sites predicted by GPS 6.0. The red letters indicate PmAGS amino acids mutated to those of SpAGS to construct the PmAGS-SpLinker mutant. **B,** Design of GFP-AGS mutant constructs tested in this study. TPR motifs are marked in blue, and GL motifs are in orange. The brown section indicates the SpAGS sequence, and the red and grey boxes show the non-sea urchin (non-SpAGS) introduced at the C-terminus. The dotted lines represent single amino acid mutations. **C¸** Single z-slice confocal images of sea urchin embryos at 8~16-cell stage showing localization of GFP-AGS-S389A mutant. Embryos were injected with 0.3μg/μl stock of GFP-AGS mRNA and 0.25μg/μl stock of 2x-mCherry-EMTB mRNA. The white arrowhead indicates vegetal cortical localization of GFP-AGS. **D,** % of the embryos with vegetal cortical localization of GFP-AGS mutants (left) and the ratio of the vegetal cortex-to-animal cortex mean intensity (right) in 16~32-cell embryos. Statistical analysis was performed against Full AGS by One-Way ANOVA. **E-F,** Embryos were injected with 0.15μg/μL stock of GFP-AGS mRNAs and 0.75mM SpAGS MO. The number of embryos forming micromeres (E) and developing to gastrula or pluteus stage (F) were scored, and each of which was then normalized to that of the Full AGS. Statistical analysis was performed by One-Way ANOVA. **G,** Design of GFP-AGS constructs tested in this study from *S. purpuratus* (Sp) and *P. miniata* (Pm). TPR motifs are marked in blue, and GL motifs are in orange. The dotted lines represent single amino acid mutations. **H¸** Single Z-slice confocal images of sea urchin embryos at 8~16-cell stage showing localization of GFP-AGS. Embryos were injected with 0.3μg/μl stock of GFP-AGS mRNAs and 0.25μg/μl stock of 2x-mCherry-EMTB mRNA. The white arrowhead indicates vegetal cortical localization of GFP-AGS. **I,** The number of embryos with vegetal cortical localization of GFP-AGS mutants in 16~32-cell embryos was scored and normalized to that of the SpAGS (left graph). Right graph shows the ratio of the vegetal cortex-to-animal cortex mean intensity. Statistical analysis was performed against SpAGS by One-Way ANOVA. **J-K,** Embryos were injected with 0.3μg/μl stock of GFP-AGS mRNAs and 0.75mM SpAGS MO. The number of embryos making micromeres (J) and developing to gastrula or pluteus stage (K) were scored and normalized to that of the SpAGS. Statistical analysis was performed by One-Way ANOVA. n indicates the total number of embryos scored. * represents p-value<0.05, ** p-value < 0.01, and *** p-value < 0.001. Each experiment was performed at least two independent times. Error bars represent standard error. Scale bars=10μm.

bioRxiv preprint doi: https://doi.org/10.1101/2024.06.30.601440; this version posted October 15, 2024. The copyright holder for this preprint (which was not certified by peer review) is the author/funder, who has granted bioRxiv a license to display the preprint in perpetuity. It is made available under aCC-BY 4.0 International license.

Supplemental Figure S5 GoLoco Domain sequence alignment

```
SpAGS_GL    461   RKGGEPIQMLDDADNFFEALSRFQSNRMDEQRCSFGRLQKKLA---------------
DmPins_GL   455   EKLRAQATRKAKDDDFFEMLSRSQSKRMDDQRCSIKVNPAGAPAVATGATRKPL----
PmAGS_GL    479   PQQRSSPEFMDDDDSFFDVLSRFQGKRMDEQRCSFNRMQDKQRERE------------
EtAGS_GL    459   QKGGEPQEMLDDADNFFEALSRFQSNRMDEQRCSFGRMQRKLQE--------------
HsLGN_GL    468   NSQRKISADTIGDEGFFDLLSRFQSNRMDDQRCCLQEKNCHTASTTTSSTPPKMMLKT
HsAGS3_GL   460   PRTSIPRAPSSDEECFFDLLTKFQSSRMDDQRCPLDDGQAGAAEATAAPTLEDRI--A
                               : **: *:: *..***:*** :

SpAGS_GL          DEEGNGLPEKEELLDEIAKLQGSRLNEQRAFSVKRLPGLPGLR-ANE---DVVGK---LL
DmPins_GL         --------------------------VQQNSLFVDPTNLPGLKSPSSANPSAIGHGPLAR
PmAGS_GL          NDEDRDYPMKEELMNQIASFQRSRLNEQRSS----ICNLPGLKTNNE---EVLGQ---LL
EtAGS_GL          DEEANGLPEKEELLDEIAKLQGSRLNEQRAFSAQRLPGLPGLR-ANE---NVLGK---LL
HsLGN_GL          SSVPVVSPNTDEFLDLLASSQSRRLDDQRAS----FSNLPGLRLTQNSQ-SVLS----HL
HsAGS3_GL         QPSMTASPQTEEFFDLIASSQSRRLDDQRAS----VGSLPGLRITHSNA-GHLR----G-
                                             *:        .****:   .     :

SpAGS_GL          AKGERAEPDDDFFEMIIRCQGARIEDQRSTLP-I--------------------------
DmPins_GL         SATTTQQPDDDFLDMLMRCQGSRLEEQRSELPRPNVTMDAEAEAPPRSVPEAAVPGAPRG
PmAGS_GL          QKGNQGVPDDDFFDMLMRCQGSRINDQRSEPP-V--------------------------
EtAGS_GL          AKGDLAVPDDDFFEMLMRCQGARIEDQRSTLP-M--------------------------
HsLGN_GL          MTNDNKEADEDFFDILVKCQGSRLDDQRCAPPPA--------------------------
HsAGS3_GL         -HGEPQEPGDDFFNMLIKYQSSRIDDQRCPPPDV--------------------------
                        .:**:::::: *.:*:::**.  *

SpAGS_GL          -QAPAPTVPDEDFFSLIQRIQSKRIEEQRSIAPWEKGSGT--------SCVCFYDYDTSR
DmPins_GL         QTGRGATVPDEDFFSLIMKVQSGRMEDQRASIPFRNANNNNNSRS-----------NNNG
PmAGS_GL          -LQPAPTVPDEDFFALIQRVQSKRMDAQRSDKGPQEQTKT--------SD----------
EtAGS_GL          -PTPAPTVPDEDFFTLIQRIQSRRIEEQRTIPPWEKGPKP--------SDK---------
HsLGN_GL          -TTKGPTVPDEDFFSLILRSQGKRMDEQRVLLQRDQNRDTDFGLKDFLQNNALLEFKNSG
HsAGS3_GL         -LPRGPTMPDEDFFSLIQRVQAKRMDEQRVDLAGGPEQGAGGPPEPQQQC-------QPG
                      . *:******:** : *. *:: **

SpAGS_GL          TEGTACCVYSRPVQESHL
DmPins_GL         SAGGAGK-----------
PmAGS_GL          ------------------
EtAGS_GL          ------------------
HsLGN_GL          KKSADH------------
HsAGS3_GL         AS----------------
```

Supplemental Figure 5. Alignment of C-terminus GoLoco domain sequences used for chimeric mutants. Sea urchin *S. purpuratus* (SpAGS_GL), *Drosophila* (DmPins_GL), sea star *P. miniata* (PmAGS_GL), pencil urchin *E. tribuloides* (EtAGS_GL), human *H. sapiens* LGN (HsLGN_GL) and human *H. sapiens* AGS3 (HsAGS3_GL). Bold letters indicate GoLoco motif sequences. The green highlight indicates additional serine amino acid present uniquely in HsAGS3_GL and mutated to Alanine in AGS_AGS3GL_3S/A mutant. The highlighted amino acids between GL2 and GL3 and within GL3 are those mutated to match HsLGN_GL in AGS_AGS3GL_GL2GL3 mutant.

bioRxiv preprint doi: https://doi.org/10.1101/2024.06.30.601440; this version posted October 15, 2024. The copyright holder for this preprint (which was not certified by peer review) is the author/funder, who has granted bioRxiv a license to display the preprint in perpetuity. It is made available under aCC-BY 4.0 International license.

Supplemental Figure 6

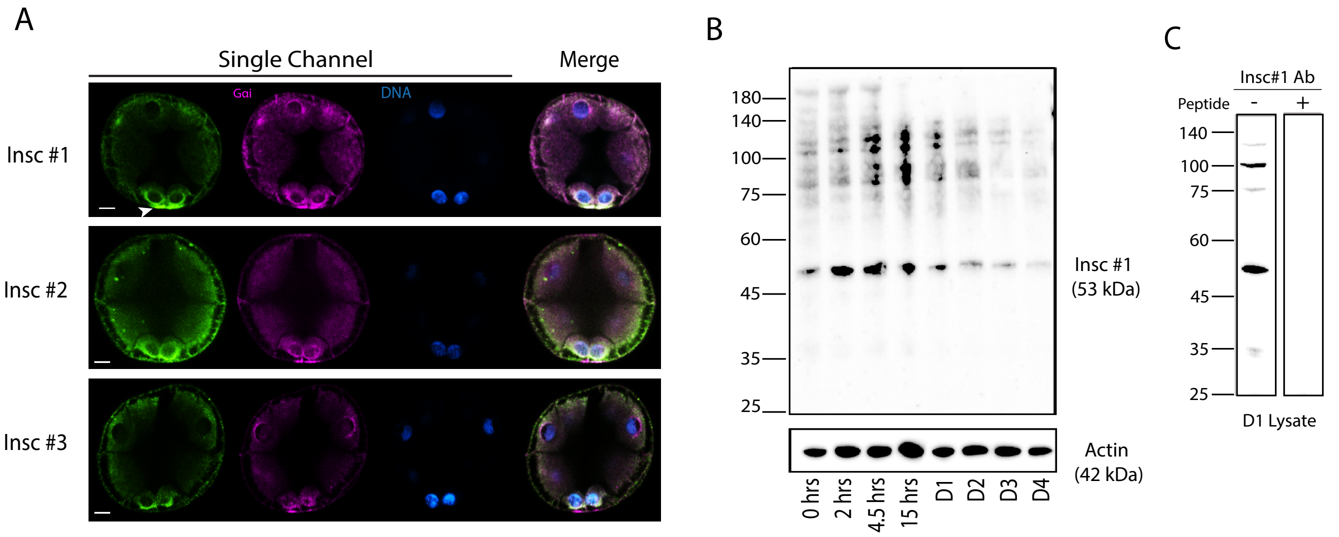

Supplemental Figure 6. Insc protein expression during embryonic development. A, Endogenous Insc protein localization by immunofluorescence. Embryos were stained with three Insc antibodies (green) designed for different Insc amino acid sequence sections. Embryos were stained with Gαi antibody (magenta) and Hoechst dye (blue). During the 16-cell stage, all antibodies show signal enriched at the vegetal pole. With #2 and #3 antibodies, some non-specific cortex signals were also observed around the entire embryo. **B**, Insc immunoblot analysis. Embryos were collected at 0, 2, 4.5, 15, 24, 48, 72, and 96 hours post fertilization and subjected to immunoblot with Insc #1 antibody. Actin (42kDa) was used as a loading control. The expected size of Insc is 53kDa. **C**, Peptide competition assay with Insc #1 antibody. The 24-hour lysate was used. Each experiment was performed at least two independent times. Scale bars=10µm.

bioRxiv preprint doi: https://doi.org/10.1101/2024.06.30.601440; this version posted October 15, 2024. The copyright holder for this preprint (which was not certified by peer review) is the author/funder, who has granted bioRxiv a license to display the preprint in perpetuity. It is made available under aCC-BY 4.0 International license.

Supplemental Figure S7

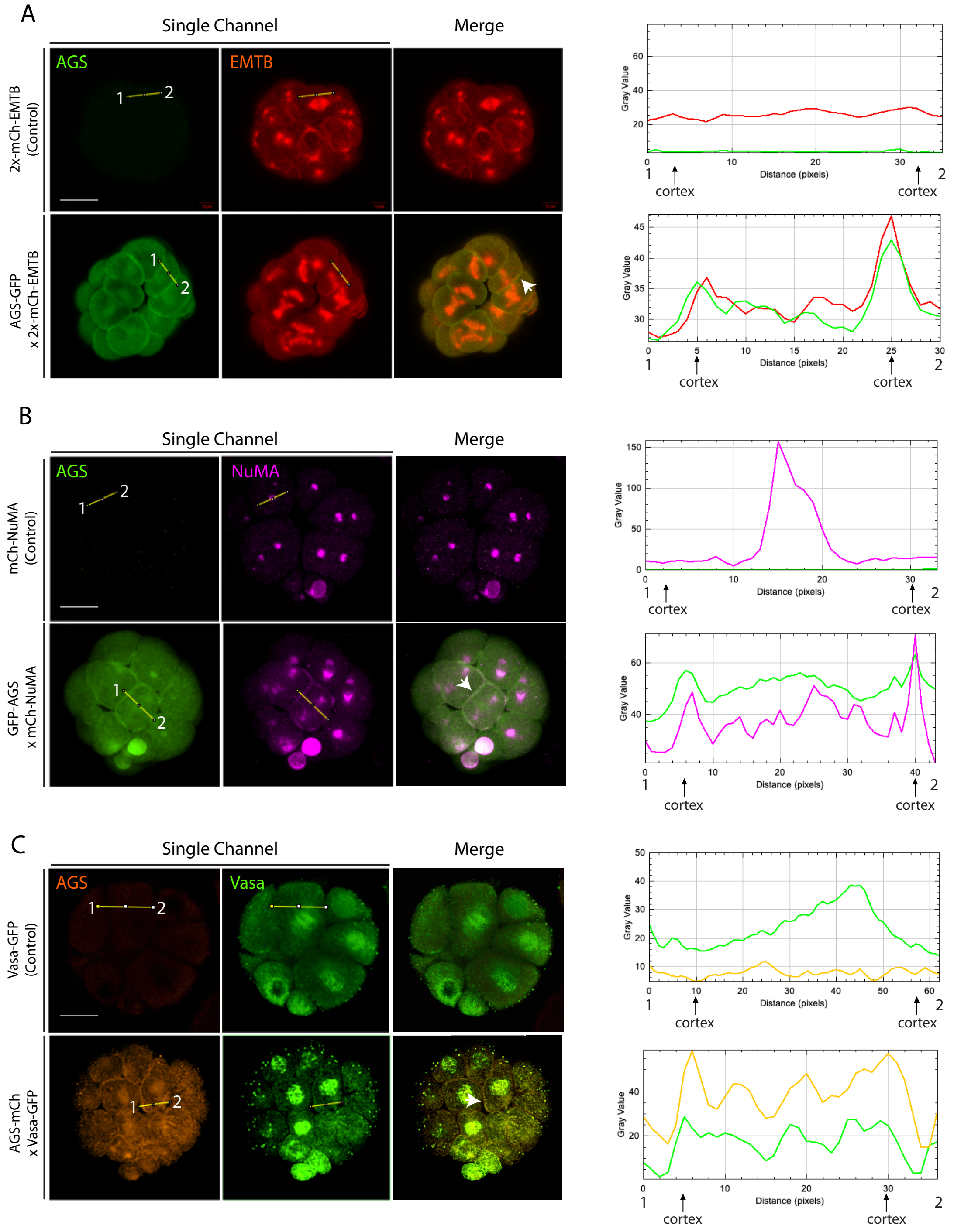

Supplemental Figure 7. SpAGS colocalizes with micromere-specific fate determinants. A, Embryos were co-injected with 2x-mCherry-EMTB (0.5µg/µl stock) mRNA with or without GFP-AGS (0.5µg/µl stock) mRNA. **B,** Embryos were co-injected with mCherry-NuMA (0.15µg/ul) mRNA with or without GFP-AGS (0.5µg/µl stock). **C,** Embryos were co-injected with Vasa-GFP (1µg/µl stock) mRNA with or without AGS-mCherry (0.5µg/µl stock) mRNA. The intensity of each signal, from one cortex to the other, was measured and plotted from point 1 to 2 on the corresponding graph (right) using *ImageJ*. White arrows indicate the cortical colocalization of each construct. All images represent over 50% of the embryos observed (n=30 or larger) per group. Scale bars=20µm.

bioRxiv preprint doi: https://doi.org/10.1101/2024.06.30.601440; this version posted October 15, 2024. The copyright holder for this preprint (which was not certified by peer review) is the author/funder, who has granted bioRxiv a license to display the preprint in perpetuity. It is made available under aCC-BY 4.0 International license.

Supplemental Figure 8 Sea urchin SpDlg and sea star PmDlg sequence alignment

```
SpDlg       ------------------------------------------------------------     0
PmDlg       MNNIEEWEYEDIILNRGGAGLGFSIAGGVDNPHVPDDPSIFITKLIPGGAAAVGGRLRLN     60

SpDlg       ------------------------------MVLRRWPRNPSTGKTLSIELIKGSKGLGFS     30
PmDlg       DVIIRVNETEIHNVPHQNAVDALKKTGSIVNLVVRRRRQRRTGDVIRIKLVKGTKGLGFS     120
                                           :: *  *:  **..: *:*:**:******

SpDlg       IAGGQGNQHVPGDNGIFVTKIIDGGAAEQDGRLQVGDKITAVGDNNLVEVTHEDAVAVLK     90
PmDlg       IAGGCGNQHVAGDNGIFVTKIIDGGAAQMDGNLQIGDKIIAVGNDKLEEVTHENAVAVLK     180
            **** ***** ****************: **.**:**** ***:::* *****:******

SpDlg       ATSNRVLLTVIKNATLLAGTQMAPYSQNDENAQPPDVSNVDMSAANPVYAT---------     141
PmDlg       ATSEVVLLTVIKGAVISYPSPPASI------TSQPDVSTMQPSSPPPPYPEAIVSSVAGV     234
            ***: *******.*.:   :  *         :. ****.:: *:  * *

SpDlg       ---MQPTLMQEPRSSDLQELDGAFGTNVAFGTNVANGANGANGSNGEPPSYNRVTNSQPP     198
PmDlg       SLQSEPAL-QRPPTPPHQEMSNMMNPPLTYHQPPQDTAYGSM---------KKMHVPPSP     284
                :*:* *.* :   **:.. :.  :::     : * *:          :::     *

SpDlg       NAFNNTPAPKIFIQNDDGIPREPRQVVLNKGATGLGFNIVGGEDGEGIFISFILAGGVAD     258
PmDlg       TANYEGNGVKPYVQNDDDFPREARTVVLNKGATGLGFNIVGGEDGEGIFISFILAGGVAD     344
            .*  :  . * ::****.:*** * ***********************************

SpDlg       LSGALRRGDQILAVNSKDLVNATHEDAALALKGAGQVVTIDAQYKPEEYNCFEAKIQSLR     318
PmDlg       LSGELKRGDQILSVNKKDLRGATHEDAALALKGTGQTVLIEAQYKPEEYNRFEAKIQSLR     404
            *** *:******:**.*** .************:**.* *:********* *********

SpDlg       EEMMQNSVS--PTGSLRTSAKRTLFVRTLFDYDKSRDSGLPSQGLSFDFGDIIHVTNASD     376
PmDlg       EEMMNSSVSSTTTGSLRTSAKRSLYVRALFDYDKTKDSGLPSQGLSFNYGDILHVTNASD     464
            ****:.***   **********:*:**:******::***********::***:*******

SpDlg       DEWWQARHILPNGEEGEIGIIPSKRRVERKEKARLRSVKFSGNKDRNNIQEPAGTVNSKG     436
PmDlg       DEWWQAKQVLPTGEEGEMGVIPSKRRVEKRERARLKSVKFSGRGGS---LESKGSMNEKR     521
            ******:::**.*****:*:********::*:***:******. .     *  *::*.*

SpDlg       KKGFF-RKFKKNRDSDQETSDPEPFTSNASDSESSYRNEEVILSYEAVVQQEIKYTRPVI     495
PmDlg       KKGFFSRKFKKGRESDLDTSDAEHITSNASDSESSYRNDEMILSYEGVVQQEVKYTRPVI     581
            ***** *****.*:** :*** * :*************:*:*****.*****:*******

SpDlg       ILGPGKDRINDDLISEMPDEFGSCVPHTTRARRPHEVDGRDYHFVESREQMEKDIQNHLF     555
PmDlg       ILGSGKDRVNDDLISEMPEKFGSCVPHTTRPRRPHEVDKRDYHFVESREQMEKDIQDHLF     641
            *** ****:*********::********** ******* *****************:***

SpDlg       IEAGQYNENLYGTSVASVKEVSEKGKHCILDVSGNAIKRLQVAQLYPIAIFLKPKSVESI     615
PmDlg       IEAGQYNENLYGTSVASVREVADKGKHCILDVSGNAIRRLQVANLHPIAIFIRPKSVESV     701
            ******************:**::**************:*****:*:*****::******:

SpDlg       VEMNRRVSEDQARKTYERALKLEQEFLEYFTAIILGDTIDDIYQQSKEIIHEQSGPTIWI     675
PmDlg       MEMNRRMSREQAEKTYERAIKLEIEFGEFFTAIISGDTLDEIYYKCKEVIHDQSGSTIWV     761
            :*****:*.:**.******:*** ** *:***** ***:*:** :.**:**:*** ***:

SpDlg       PAKEKL    681
PmDlg       PAKDKL    767
            ***:**
```

PDZ
SH3
GUK

Supplemental Figure 8. Sea urchin (SpDlg) and sea star (PmDlg) sequence alignment. Blue, yellow, and green highlights indicate the PDZ, SH3, and GUK domains, respectively.

bioRxiv preprint doi: https://doi.org/10.1101/2024.06.30.601440; this version posted October 15, 2024. The copyright holder for this preprint (which was not certified by peer review) is the author/funder, who has granted bioRxiv a license to display the preprint in perpetuity. It is made available under aCC-BY 4.0 International license.

Supplemental Figure 9 Sea urchin SpInsc, pencil urchin EtInsc and sea star PmInsc sequence alignment

```
PmInsc      ----------MNGFNES-ALAGWLADDVIEMHMTDEDSVVRWLQDLRTMTECECMCILQG      49
SpInsc      ----------MSDN----KNTESFP-EKKLMQRCDVDSVQQWLIDIRLMPEQECTTVMET      45
EtInsc      MTCLYGVVRTMYGPQAPVICEEKLPTSTSRFQLTEVRSVQQWLSDLQGMMECECN-ILET      59
                      * .          :  .   ::  :  ** :** *:: * * **  :::

PmInsc      KPIRTSAHDMAKVTSSARDNIYILRQRASSISADFAKLYYKLDSGSWDQVH-WLCLSLTI      108
SpInsc      KPIHNSPLNLENLAATVKESVYRLQSIKDGLFSDLSQIYMCLDLSDQ------------      92
EtInsc      KPIHNSPLNLENVAAATKENIYRLRNRAENLARELQQLYHKLTSGTYSQRLRPAVLALTL      119
            ***:.*  :: :::::.::.:* *:.  ..:  :: ::*  *  .

PmInsc      RLRTFLHECTSYVPTMPLHLFQRQQTVLEECGRLVKHAESFEELNGTLPPALPTLNVLSS      168
SpInsc      ------------------------------------------------SVK--LFIQSLTT      103
EtInsc      HVRSCLEECNEYCPDF-LHMKEKQQVVIDAMATLVKNFEMYDNSKGCELD--SLIHSLTS      176
                                                                  :: *::

PmInsc      LGKAFNDLVHMVQGILIQTVVRSIHESASEYNIKIAINSIHCLSEDDSEIRRLLAKEGAI      228
SpInsc      VAKAIKAYLEMVQGALVRKLVDMVQGAATELNVKFAVLTVHSLAQDGVWLRRLLIQEDTM      163
EtInsc      LGNALNSIRQTVQSALVRKLVMMIQDSASELNVKFAVITAHSLSQDSIWLRRILIQENAM      236
            :.:*::   . **. *::.:*  :: :*:* *:*:*: : *.*::*.  :**:* :*.::

PmInsc      QALLQLCQQASL--NAMKPMALKTVANLCNVAEGRQELERASGVEYLSEILSSPTATESV      286
SpInsc      RALLAICRITTFSIRSIPMTALEIVSILCTEVEGRAELEKVGGVGALSEILASPITLEAV      223
EtInsc      GALFAVCRITTLSIPSIVTTALQAISTLCAEPEGRVELEKIGGLGFLGEILSSPSTSEAV      296
             **: :*: :::   ::   **: :: **   *** ***: .*:  *.***:** : *:*

PmInsc      KAEAASVIAQITSPNIDNYHHLLGFIEYMEDLLKDLTALSGSASSSSVFVIATSAIANIT      346
SpInsc      KKEAASVLAEITSPDADIFHRMLCFIEHMEDLLRNLTVLCDQTSSPGVFLVATTAIANIT      283
EtInsc      KLEAASVLAEITSPDINSYHRMLCFIEHMEDLLKNLTALCDQTSNPLVFLVSASAIANIT      356
            * *****:*:****: : :*::* ***:*****::**.*...:*.  **:::::******

PmInsc      FMDSMACDCLASFHTARALVKGCNNGMANTLYCKDQVATILANMAVTDQCRKEIMVSGGT      406
SpInsc      FMDAMASDYLAEFQTAEVLIQGYLKGKAPSIYSKHQVITIIANISCSKSCREQIVSSGGM      343
EtInsc      FMDTMSSDFLAMFNTATILIDGYTSGKAQNMYSKDQVVTIIANMSGSEKCREQIIKAGGM      416
            ***:*:.* ** *:**  *:.*  .* * .:*.*.** **:**:: :..**::*: :**

PmInsc      ELLVDFLHTRALVHPSSAK---KGLKGSGLSGGCSSGGKQDVVMSSSCEKVHQKAAIALA      463
SpInsc      NILAELLQGVPSTSRPTSLSRKLSFDGN--------VSLGQMGEESAYEDIYQKAATALG      395
EtInsc      KVLVELLHTRAPFTTVKTNGVN---QRA--------DSKAACGMASTCEHVHQKSAIALG      465
            ::*.::*:        .:       .           .       *: *.::**:* **.

PmInsc      RLARDQDSAELILELDAIPRLVELCCNRRERNDSEPVLVACLAALRKLHNLLGPSVFKSS      523
SpInsc      RLCQDYETCKMAVDLKVVSRLADLCKHARERNHRSDVLVACLAALRRIHSHIGSSPLKEN      455
EtInsc      RLCQDHDTAMMAVDLGVIPRLVDMCRHRRERNDSEPVLVACLAALRKLHSVVGASKFEPK      525
            **.:* ::. : ::* .: **.::* : ****. . **********::*. :* * :: .

PmInsc      DVDQLIKPRLIDSYLMCSATK--ESFV  548
SpInsc      DVEQLIQPRLMESFLHCSTTVRRETFV  482
EtInsc      ALQQLIRPRLIESFVTCSSIGSTETFV  552
             ::***:***::*:: **:    *:**
```

LBD (LGN-binding domain)

Supplemental Figure 9. Sea urchin (SpInsc), pencil urchin (EtInsc), and sea star (PmInsc) sequence alignment. The blue highlight indicates the LBD domain.
